# A Trivalent Enzymatic System for Uricolytic Therapy of HPRT Deficiency and Lesch-Nyhan Disease

**DOI:** 10.1007/s11095-017-2167-6

**Published:** 2017-05-15

**Authors:** Luca Ronda, Marialaura Marchetti, Riccardo Piano, Anastasia Liuzzi, Romina Corsini, Riccardo Percudani, Stefano Bettati

**Affiliations:** 1 Department of Medicine and Surgery,, University of Parma, Parco Area delle Scienze 23/A, 43124 Parma, Italy; 2 Department of Chemistry, Life Sciences, and Environmental Sustainability, University of Parma,, Parco Area delle Scienze 23/A, 43124 Parma, Italy; 3 National Institute of Biostructures and Biosystems, Viale Medaglie d’Oro 305, 00136 Rome, Italy

**Keywords:** enzyme therapy, gout, hyperuricemia, Lesch-Nyhan disease, polyethylene glycol, purine degradation

## Abstract

**Purpose:**

Because of the evolutionary loss of the uricolytic pathway, humans accumulate poorly soluble urate as the final product of purine catabolism. Restoration of uricolysis through enzyme therapy is a promising treatment for severe hyperuricemia caused by deficiency of hypoxanthine-guanine phosphoribosyltransferase (HPRT). To this end, we studied the effect of PEG conjugation on the activity and stability of the enzymatic complement required for conversion of urate into the more soluble (*S*)-allantoin.

**Methods:**

We produced in recombinant form three zebrafish enzymes required in the uricolytic pathway. We carried out a systematic study of the effect of PEGylation on the function and stability of the three enzymes by varying PEG length, chemistry and degree of conjugation. We assayed *in vitro* the uricolytic activity of the PEGylated enzymatic triad.

**Results:**

We defined conditions that allow PEGylated enzymes to retain native-like enzymatic activity even after lyophilization or prolonged storage. A combination of the three enzymes in an appropriate ratio allowed efficient conversion of urate to (*S*)-allantoin with no accumulation of intermediate metabolites.

**Conclusions:**

Pharmaceutical restoration of the uricolytic pathway is a viable approach for the treatment of severe hyperuricemia.

**Electronic supplementary material:**

The online version of this article (doi:10.1007/s11095-017-2167-6) contains supplementary material, which is available to authorized users.

## Introduction

**Fig. 1:**
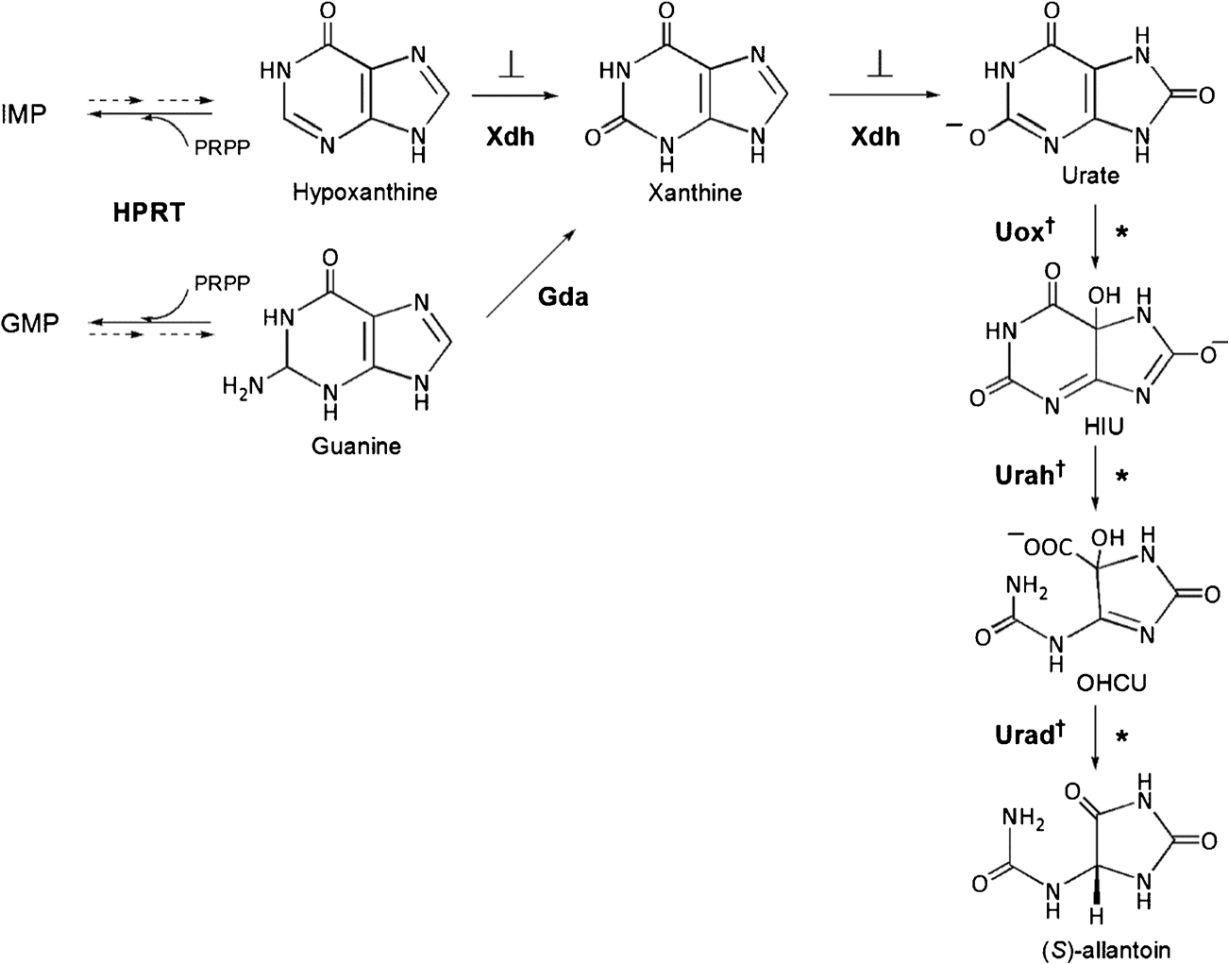
Urate biosynthesis and degradation. Enzymes names are in bold, and abbreviated as follows: *HPRT* hypoxanthine-guanine phosphoribosyltransferase, *Xdh* xanthine oxidase/dehydrogenase, *Gda* guanine deaminase, *Uox* urate oxidase, *Urah* 5-hydroxyisourate (HIU) hydrolase, *Urad* 2-oxo-4-hydroxy-4-carboxy-5-ureidoimidazoline (OHCU) decarboxylase. Genes that are lost in hominoids are denoted by a dagger symbol. Reactions inhibited by allopurinol and febuxostat are indicated by inhibition symbols. The proposed therapeutic approach is based on the facilitation of purine degradation through the administration of uricolytic enzymes (*asterisks*), and is anticipated to normalize the level of upstream metabolites.

Different from hominoids, most mammals do not show gout or urate kidney stones formation as they are able to convert the uric acid to its much more soluble metabolite (*S*)-allantoin. It has been recently reported that urate oxidase (uricase, Uox) is not the unique enzyme involved in this process (1–3). Phylogenetic genome comparison highlighted two other genes sharing with urate oxidase evolution events, encoding enzymes catalyzing two further steps following urate oxidation to 5-hydroxyisourate (HIU): hydrolysis of HIU to give 2-oxo-4-hydroxy-4-carboxy-5-ureidoimidazoline (OHCU), catalyzed by HIU hydrolase (Urah), and decarboxylation of OHCU to give (*S*)-allantoin, catalyzed by OHCU decarboxylase (Urad) (Fig. 1). Although HIU can spontaneously degrade to racemic allantoin through other intermediates on a time scale of several hours, the metabolic pathway constituted by the three enzymes directly produces (*S*)-allantoin in seconds.

Excess of serum urate (hyperuricemia) increases the risk of gout and metabolic comorbidities such as obesity, hypertension, and diabetes (4–6). The current pharmacological approaches to hyperuricemia-related diseases exploit competitive inhibitors of xanthine oxidase (e.g. allopurinol and febuxostat), inhibitors of the renal urate transporters (e.g. probenecid), or the intravenous administration of recombinant Uox. Two different formulations of the latter have been approved for human use in US and Europe: Rasburicase (Elitek®, Sanofi-Aventis, for the prevention and treatment of tumor lysis syndrome) (7) and Pegloticase (Krystexxa®, Savient Pharmaceuticals, for treatment-refractory, chronic gout) (8,9).

We recently proposed that the treatment of severe early onset hyperuricemia, as observed in hereditary hypoxanthine-guanine phosphoribosyltransferase (HPRT)-deficiency and Lesch-Nyhan disease (LND), could be approached by facilitating rather than blocking the purine degradation pathway (uricolytic *versus* uricostatic approach). When purine salvage is impaired, as in the case of HPRT deficiency, uricostasis causes the accumulation of urate precursors xanthine and hypoxanthine (Fig. 1). Excess of hypoxanthine has been implicated in the severe neurological manifestations of LND (10), while xanthine urolithiasis and renal failures are observed in LND patients treated with allopurinol (11,12). In addition, cases have been reported of LND patients that are irresponsive or intolerant to uricostatic drugs (13).

In the organisms in which the urate degradation pathway is preserved, all three enzymes responsible for the degradation of urate to allantoin (Uox, Urah and Urad) are invariantly present. This suggests an evolutionary pressure to avoid the accumulation of intermediates of the urate oxidation pathway. While currently approved uricolytic treatments rely on the administration of Uox alone (Rasburicase or Pegloticase), we aim at implementing a prolonged therapy of severe hyperuricemia with the full set of uricolytic enzymes. The Uox-Urah-Urad enzymatic triad would lower the concentration of urate oxidation products, known to be toxic in the animal model (14), by catalyzing rapid conversion of urate into a non-toxic easily excreted metabolite. We have previously obtained recombinant Urah and Urad from mouse (2) and zebrafish (15,16). For the purpose of this study, we selected the zebrafish (*Danio rerio*) enzymes because of the availability of three-dimensional structures (15,16), and the higher solubility and stability of the isolated proteins. We recently completed the enzymatic set by obtaining recombinant Uox from the same source (17). To improve the bioavailability of *Dr*Uox, *Dr*Urah and *Dr*Urad we used chemical conjugation with polyethylene glycol (PEG), a well-known technology that has been used to prolong half-life of biotech-drugs (18,19). PEG is a biocompatible polymer that can be covalently conjugated to proteins to improve their biocompatibility and reduce immunological recognition and clearance (19–23). Although there are alternative strategies of surface chemical modification and protein engineering that could improve bioavailability and antigenic masking (24–26), we selected PEG because so far the about a dozen bioconjugate therapeutics in clinical use (including Pegloticase) are based on this technology (19,24,27,28). In addition, the functional relevance of induced anti-PEG antibodies has been recently challenged, based on a critical review of the existing literature (29–32).

We focused our PEGylation strategy on reactive thiols as anchoring points for maleimido-functionalized PEG (MAL-PEG) derivatives, also exploiting extension arm-facilitated PEGylation chemistry (33). We systematically studied of the effect of PEG molecular weight and degree of conjugation on the retention of enzyme activity before and after lyophilization. Each of the three PEGylated, recombinant enzymes was obtained in an active form. The enzymatic triad was tested *in vitro* and shown to catalyze full conversion of urate to (*S*)-allantoin, at variance with the use of Uox alone, that causes a significant accumulation of the HIU metabolite. These results indicate that restoration of a complete uricolytic pathway by this enzymatic triad can be applied without affecting the activity of all three enzymes, thus opening the way to future tests in suitable animal models currently under development in our laboratories.

## Materials and Methods

### Chemicals

All chemicals were of the best commercial quality available and were used without further purification. Xanthine-agarose resin was purchased from Sigma-Aldrich. Polyethylene glycol functionalized with maleimido–group (MAL-PEG) with nominal molecular weight of 5 kDa was purchased from NOF Corporation. 20 kDa and 40 kDa MAL-PEG were obtained from Iris Biotech GMBH. Millipore Amicon Ultra ultrafiltration tubes (with membrane cut-off of 30 kDa and 100 kDa) were supplied by Merck Millipore. Micro Float-A-Lyzer® micro dialysis devices (cut-off membrane 20 kDa and 50 kDa) were purchased from Spectrum Labs.

### Enzymes Expression and Purification

A pET11b-derived plasmid encoding wild type urate oxidase from zebrafish, and pET28b-derived plasmids encoding HIU hydrolase and OHCU decarboxylase (*Dr*Uox, *Dr*Urah and *Dr*Urad, respectively) (15–17), were transformed into *E. coli* BL21 CodonPlus (DE3)-RIL cells (Merck-Millipore). For *Dr*Urad, before cell transformation the amplified sequence was sub-cloned into pET28b expression vector (Novagen) exploiting NdeI and BamHI restriction sites in frame with N-terminal poly-histidine molecular tag coding sequence. Cells were grown at 37 °C in LB medium supplemented with the appropriate antibiotics. At OD_600 nm_ ≈ 0.6–0.9, IPTG was added to the media to a final concentration of 1 mM. The cultures were further incubated at 20 °C for 20 h or 4 h in the case of *Dr*Uox and *Dr*Urad, respectively, and at 28 °C for 4 h in the case of *Dr*Urah. Cells were harvested by centrifugation and resuspended in lysis buffer (*Dr*Uox and *Dr*Urah: 50 mM sodium phosphate, 300 mM NaCl, 10% v/v glycerol, 200 μM PMSF, 200 μM benzamidine, 1.5 μM pepstatin at pH 8.0; *Dr*Urad: 50 mM sodium phosphate, 300 mM NaCl, 10% v/v glycerol, 1 μM pepstatin, 1 μM leupeptin, 100 μM PMSF at pH 8.0).

The suspensions were then incubated at 4 °C for 45 min under agitation in the presence of 1 mg/ml lysozyme, with the exception of *Dr*Urah. Cells were then sonicated (20 s for each cycle, 1 s ON and 1 s OFF, with pauses of one minute between cycles) and the lysate was centrifuged at 4°C for 30 min at 16000 g.

*Dr*Uox was further purified by affinity chromatography onto an agarose resin functionalized with xanthine (Sigma-Aldrich), an analog of the enzyme substrate that cannot be oxidized by Uox (34,35). The protein was eluted by adding a fresh 0.5 mM uric acid solution in 100 mM potassium phosphate, pH 7.6. *Dr*Urah was purified by two subsequent chromatographic steps: a cationic exchange chromatography in 50 mM MES buffer, pH 5.9 was carried out on a 5 ml SP FF column (GE Healthcare), followed by an anionic exchange chromatography in 50 mM CHES buffer, pH 9.5 on a 5 ml Q FF column (GE Healthcare). *Dr*Urad, containing an N-terminal poly-histidine tag, was purified by affinity chromatography on cobalt resin (Talon, Clontech) pre-equilibrated with a 50 mM sodium phosphate solution, pH 8.0, containing 300 mM NaCl and 20 mM imidazole. The column was washed with the same buffer and the protein was eluted with 50 mM sodium phosphate buffer, pH 8.0, 300 mM NaCl and 200 mM imidazole. The three proteins solutions were finally diafiltered with a solution containing 100 mM potassium phosphate, 150 mM NaCl, 5% v/v glycerol, pH 7.6, and concentrated by Amicon Ultra-15 centrifugal filter devices (Merck-Millipore), with 100 kDa cut-off for *Dr*Uox and 30 kDa cut-off for *Dr*Urah and *Dr*Urad. *Dr*Uox was also filtered with a 0.2 μm filter unit to eliminate possible protein aggregates. All enzymes were pure to near homogeneity, as indicated by densitometric analysis of SDS-PAGE which did not detected any measurable amount of other protein components. The solutions of the three enzymes were divided in small aliquots, flash-frozen in liquid nitrogen and stored at −80°C.

### PEGylation of Enzymes

The reactions were carried out in vials at 20°C, by adding fresh reagents solutions to a 2 mg/ml *Dr*Uox, *Dr*Urah or *Dr*Urad protein stock solution, in a solution containing 100 mM potassium phosphate, 150 mM NaCl, pH 7.4. The reaction of MAL-PEG with the cysteine side chains of *Dr*Uox or *Dr*Urah was stopped by the addition of a molar excess of free cysteine. With 5 kDa PEG, two different MAL-PEG/free cysteines molar ratios were sampled: 1.25/1 and 7.5/1. The degree of protein PEGylation was checked by SDS-PAGE at 15, 30 and 45 min. Reaction with 20 kDa and 40 kDa PEG was carried out at a 7.5/1 MAL-PEG/free cysteines molar ratio, with a 30 min incubation time.

For *Dr*Urad, an extension arm-facilitated PEGylation protocol (33) was also carried out (using 2- iminothiolane and MAL-PEG). The enzyme was incubated at 20°C with 2-iminothiolane (IMT) for five minutes, followed by MAL-PEG addition, left to react for ten minutes. The total reaction took 15 min. Both reactions were quenched by the addition of an excess of lysine and cysteine. With 5 kDa MAL-PEG, the conditions sampled included 20x or 40x molar excess of IMT with respect to protein concentration, and a MAL-PEG/*Dr*Urad molar ratio of 2.5, 5 or 10. When the PEGylation reaction was carried out with 20 kDa or 40 kDa MAL-PEG only a 20x molar excess of IMT with respect to protein concentration and a MAL-PEG/*Dr*Urad molar ratio of 5 were used.

After quenching the PEGylation reaction, the samples were dialyzed at 4 °C in 100 mM potassium phosphate, 150 mM NaCl, pH 7.6, in order to eliminate unreacted MAL-PEG and other reagents. For *Dr*Urad, protein samples were dialyzed with Micro Float-a-Lyzer® devices (Spectrum Labs).

### SDS-PAGE of PEGylated Enzymes

Protein samples were prepared by precipitation with acetone followed by pellet drying and resuspension in sample buffer. Staining was carried out with Biosafe® Coomassie for protein detection and barium iodide for PEG detection. The SDS-PAGE gels were scanned using a ChemiDoc® imager (Biorad) and relative bands intensity was evaluated by densitometric analysis. At least two different protein concentrations were tested to verify that sample loading was within linearity range.

### Activity Assays

The activity assays for the three enzymes were performed at 37°C in 100 mM potassium phosphate, pH 7.4. The enzymatic activity of *Dr*Uox was analyzed by directly monitoring at 292 nm the uric acid consumption in the presence of 100 μM urate and at least 0.07 μM HIU hydrolase. The secondary enzyme is required in order to eliminate the reaction product HIU, which absorbs in the same UV region of urate. The reaction was started by adding Uox to the reaction solution.

The unstable substrates HIU and OHCU were produced *in situ* from urate, by adding the enzymes responsible of their synthesis. The activity of *Dr*Urah and *Dr*Urad was monitored by measuring absorbance at 312 nm and 257 nm, respectively, in the presence of at least 0.8 μM *Dr*Uox (for *Dr*Urah), 0.8 μM ﻿ *Dr*Urah (for *Dr*Urad) and 100 μM urate, as previously reported (2,15). The production of the substrate was started in both cases by adding Uox to the reaction. When the maximum of absorbance was reached (i.e., all the urate was converted into HIU or OHCU, respectively) *Dr*Urah or *Dr*Urad were added to the assay solution.

### Lyophilization

The PEGylated enzymes and the control samples were split in small aliquots and 4% mannitol and 1% sucrose (w/v, final concentrations) were added as lyoprotectant agents (36). All the aliquots were then flash-frozen in liquid nitrogen and successively lyophilized O/N using a Modulyo® Freeze Dryer apparatus (Edwards). Lyophilized samples were then stored at −20 °C.

### Circular Dichroism Spectroscopy

Circular dichroism spectra were recorded with a Jasco J715 spectropolarimeter equipped with a Peltier thermostatic cell set at 20 °C. Spectra were collected using a 2 mm quartz cuvette, in 20 mM potassium phosphate, pH 7.4. Spectra were recorded in the far-UV region, between 195 and 260 nm, to probe the secondary structure of proteins; e.g., negative ellipticity bands at 222 nm are typical of alpha-helical elements, and their intensity is a measure of protein alpha-helical content. Since all asymmetric molecules are optically active in the region of the spectrum where they absorb light, i.e. they provide a circular dichroism signal, spectra in the 200–340 nm wavelength range were recorded to measure urate conversion to HIU and OHCU.

## Results

### Enzymes PEGylation Strategy

Among the different established methods for protein PEGylation (19–22,37), we selected an approach based on the three-dimensional structure of the target proteins (Fig. S1). *Dr*Uox is a homotetramer, as invariantly found for all known uricases. *Dr*Urah is also a homotetramer, while *Dr*Urad is a homodimer. The oligomeric organization of these three enzymes is evolutionarily conserved both in eukaryotes and prokaryotes. In the case of *Dr*Uox and *Dr*Urah the active site is formed at the subunit interface, indicating that the protein monomer is not catalytically functional. Inspection of the sequence and structure of the three enzymes shows that 92 lysine residues are present in the *Dr*Uox tetramer, 12 in the *Dr*Urah tetramer, and 20 in the *Dr*Urad dimer. In *Dr*Uox, two conserved lysine residues (K18 and K159) are involved in catalysis or substrate binding (17); however, analysis of accessible surface area (ASA) suggests that these residues could not be accessible. According to ASA analysis, 84, 12, and 20 lysine side chains are partially or fully accessible at the *Dr*Uox, *Dr*Urah, and *Dr*Urad surface, respectively (Supplementary Table S1). Although amine-reactive chemistry rarely modifies all available lysine residues on the surface of a protein, we were concerned about the possibility to control sample homogeneity. Moreover, the reaction of lysine to form an amide bond will reduce the net charge and change the pI of the protein (non-conservative PEGylation). Based on the above considerations, we decided to exploit the reactivity of reduced cysteines, constitutively present on the three enzymes, by using maleimido-PEG (MAL-PEG). MAL-PEG directly reacts with the thiol groups exposed at the protein surface, and the reaction does not change the number of charges of the molecule (conservative PEGylation). This PEGylation strategy already yielded FDA approved antibody-drug conjugates (Brentuximab vedotin and Trastuzumab emtansine) and a PEG-conjugate (Certolizumab pegol, Cimzia), demonstrating that this chemistry is suitable for in vivo administration.

Cysteine residues have not been implicated in the catalytic activity of the three zebrafish enzymes (15–17). *Dr*Uox, *Dr*Urah and *Dr*Urad oligomers contain 20, 8, and 10 cysteine residues, respectively, of which 4, 4 and 2 are at least partially accessible (Supplementary Table S2). Visual inspection of the *Dr*Uox structure (Fig. S1) indicates that under oxidizing conditions 4 Cys side chains of the tetramer are engaged in disulfide bridges and 16 are available for chemical conjugation. Therefore, preliminary optimization of the PEGylation reactions passed through the evaluation of the reactivity of the available cysteines for each of the three enzymes. Although the high cysteine to protein ratio made a reliable quantification of the reactive cysteines by using Ellman’s reagent difficult, a significant number of PEGylation sites with high and similar reaction efficiency was found for the three proteins. This property is considered a positive condition for obtaining a homogeneous protein formulation, expected to promote a more homogeneous composition of the conjugate (38).

### Effect of PEG Size and Degree of Conjugation on Enzyme Activity and Stability

#### PEGylation of *Dr*Uox

In order to optimize reaction conditions, we initially investigated the time-evolution of the reaction of zebrafish urate oxidase (*Dr*Uox) with 5 kDa MAL-PEG, at two different PEG/free cysteines molar ratios (1.25 and 7.5).

**Fig. 2:**
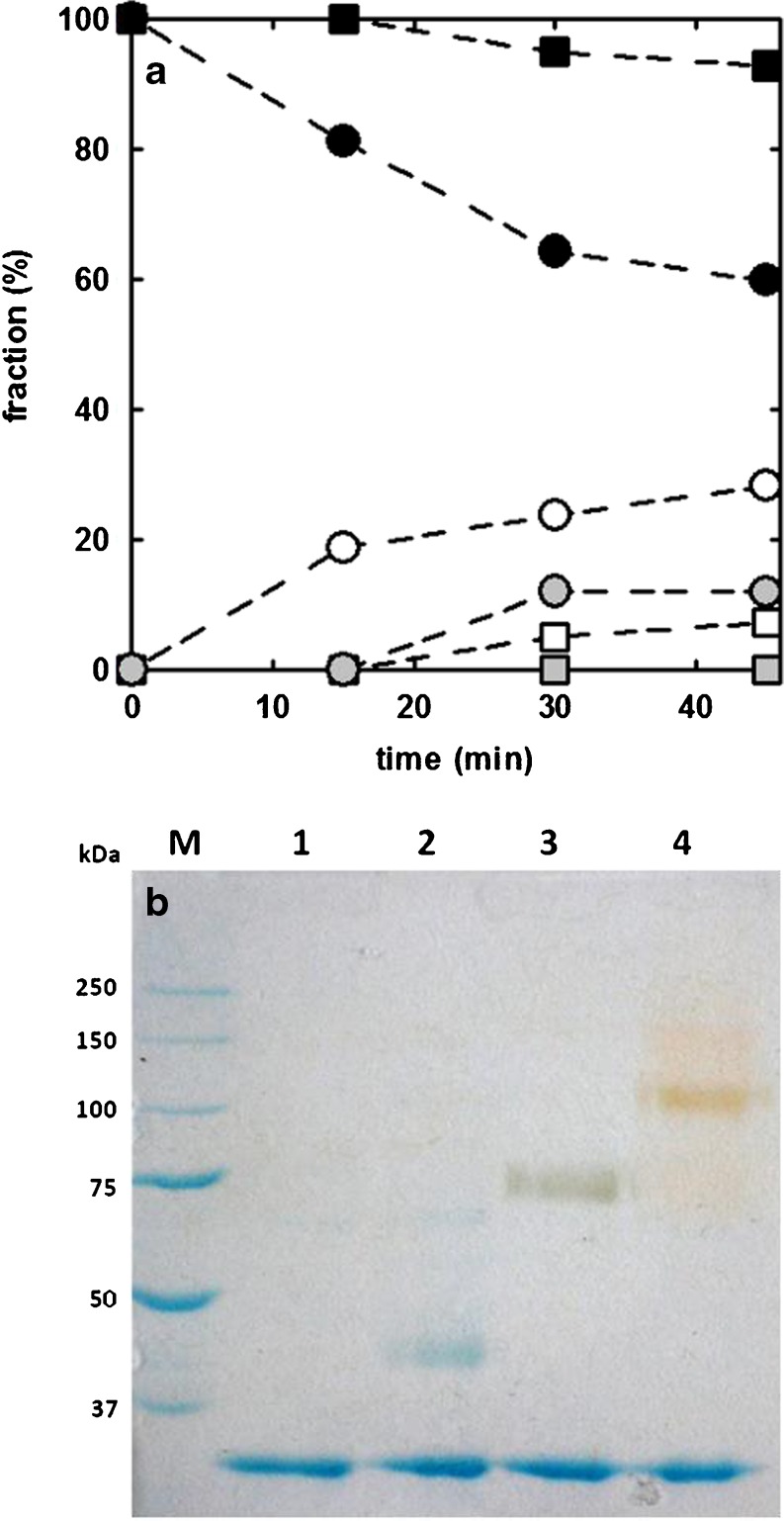
PEGylation of *Dr*Uox. Panel (**a**) PEGylation kinetics at different PEG/cysteine molar ratios. The conjugation reaction was carried out at 20 °C, with 5 kDa MAL-PEG, at a MAL-PEG/reactive cysteines molar ratio of 7.5 (*circles*) and 1.25 (*squares*). *Grey symbols* refer to the fraction of di-PEGylated monomers, open symbols to the fraction of mono-PEGylated monomers, closed symbols to the fraction of unmodified monomers. Dashed lines through data points are just drawn for eye guidance. Panel (**b**) SDS-PAGE gel of unmodified and PEGylated *Dr*Uox (PEG/cysteine molar ratio of 7.5). *Lane M*, MW standards; *Lane 1*, unmodified *Dr*Uox; *Lane 2*, *Dr*Uox + 5 kDa PEG; *Lane 3*, *Dr*Uox + 20 KDa PEG; *Lane 4*, *Dr*Uox + 40 kDa PEG. The gel underwent a double staining procedure, first with Coomassie blue (*blue bands*), and then with the PEG-specific dye barium iodide (*yellow-brown bands*).

Figure 2a reports the results of densitometric analysis of SDS-PAGE gel bands (Fig. S2), indicating that the degree of conjugation strongly depends on PEG molar excess, as expected, and that the conjugation reaction does not reach completion within the observed time window.

Considering the modest progression of the conjugation reaction at times longer than 30 min, and the necessity to assure a sufficient degree of PEGylation, while limiting the incubation time for concerns relative to protein stability at room temperature, a 7.5 PEG/cysteine molar ratio and a 30 min reaction time were finally chosen and kept constant for all following conjugation reactions on *Dr*Uox.

Although other features may be involved, including sites and degree of conjugation, the stability of PEGylated proteins positively correlates with PEG molecular weight, as in the case of trypsin (39) and α-chymotrypsin (40), and it was reported that the molecular weight of linear or branched PEG may affect protein activity and clearance time in vivo (41). The effect of PEG chain length on the enzymatic activity of *Dr*Uox was assessed upon conjugation with 5, 20 and 40 kDa MAL-PEG.

Figure 2b reports SDS-PAGE of *Dr*Uox reacted with 5, 20 and 40 kDa MAL-PEG, in comparison with the unmodified protein. PEGylation with MAL-PEG of different molecular weight always resulted in a significant protein derivatization. Yellow-brown bands in lanes 2, 3 and 4 are due to gel staining with a dye specific for PEG (barium iodide). Barium iodide staining was carried out after Coomassie blue staining to confirm the attribution of slowly migrating bands to PEGylated forms of *Dr*Uox monomers. The protein bands of the gel underwent densitometric analysis to evaluate the degree of PEGylation of single monomers. The results of densitometric analysis were then used to calculate the average number of PEG molecules bound per biological unit (the tetramer, in the case of Uox and Urah, or the dimer, in the case of Urad), and the percentage of unmodified tetramers (i.e. tetramers containing 4 unmodified protein subunits) (Table I). The calculation was carried out by means of a combinatorial procedure that we previously reported for PEGylated hemoglobin (42). The results show that i) *Dr*Uox PEGylation with MAL-PEG of different molecular weight resulted in similar PEGylation yields, and ii) despite the relative abundance of non-PEGylated monomers, after quaternary assembly only a low percentage of unmodified tetramers occurs (Table I). The lack of an evident decrease in conjugation efficiency with the increase of PEG MW likely depends on the low degree of functionalization (no more than two PEG chains per monomer): only at higher derivatization rates we would expect to see the steric hindrance effect of PEG chains attached to protein surface.

**Table I:** PEGylation Yield and Enzymatic Activity of DrUox after Reaction with MAL-PEG (PEG/Cysteine Molar Ratio of 7.5)

	% non-PEGylated monomers ^a^	% mono-PEGylated monomers ^a^	% di-PEGylated monomers ^a^	Average no. of PEG bound (per tetramer) ^b^	% unmodified tetramers ^b^	Specific enzymatic activity ^c^
No PEG	100	0	0	0	100	7.8 ± 0.2
5 kDa PEG	60	28	12	2.1	13	7.8 ± 1.2
20 kDa PEG	46	54	0	2.2	5	11.1 ± 0.6
40 kDa PEG	71	21	8	1.5	25	12.6 ± 0.1

^a^determined from densitometric analysis of SDS PAGE

^b^calculated from combinatorial analysis

^c^specific enzymatic activity is expressed as μmol min^−1^ mg^−1^ (mean ± standard error). The specific enzymatic activity of freshly prepared enzyme was 8.9 ± 0.9

The enzymatic activity of all samples was checked right after the conjugation reaction. The results, shown in Table I, indicate a good retention of the enzymatic activity after PEGylation. Activity was indeed very close to the native enzyme when the derivatization was carried out with 20 kDa or 40 kDa PEG. The preservation of activity cannot be attributed to the very low fraction of unmodified protein (5% of non-PEGylated tetramers in the case of conjugation with 20 kDa PEG). The enzymatic activity of the unconjugated protein was measured on samples that underwent the same procedure of PEGylated ones, except that MAL-PEG was not included in the reaction mixture. The value slightly lower than that of freshly prepared enzyme is consistent with the observation that recombinant *Dr*Uox is marginally stable at room temperature.

#### PEGylation of *Dr*Urah

**Fig. 3:**
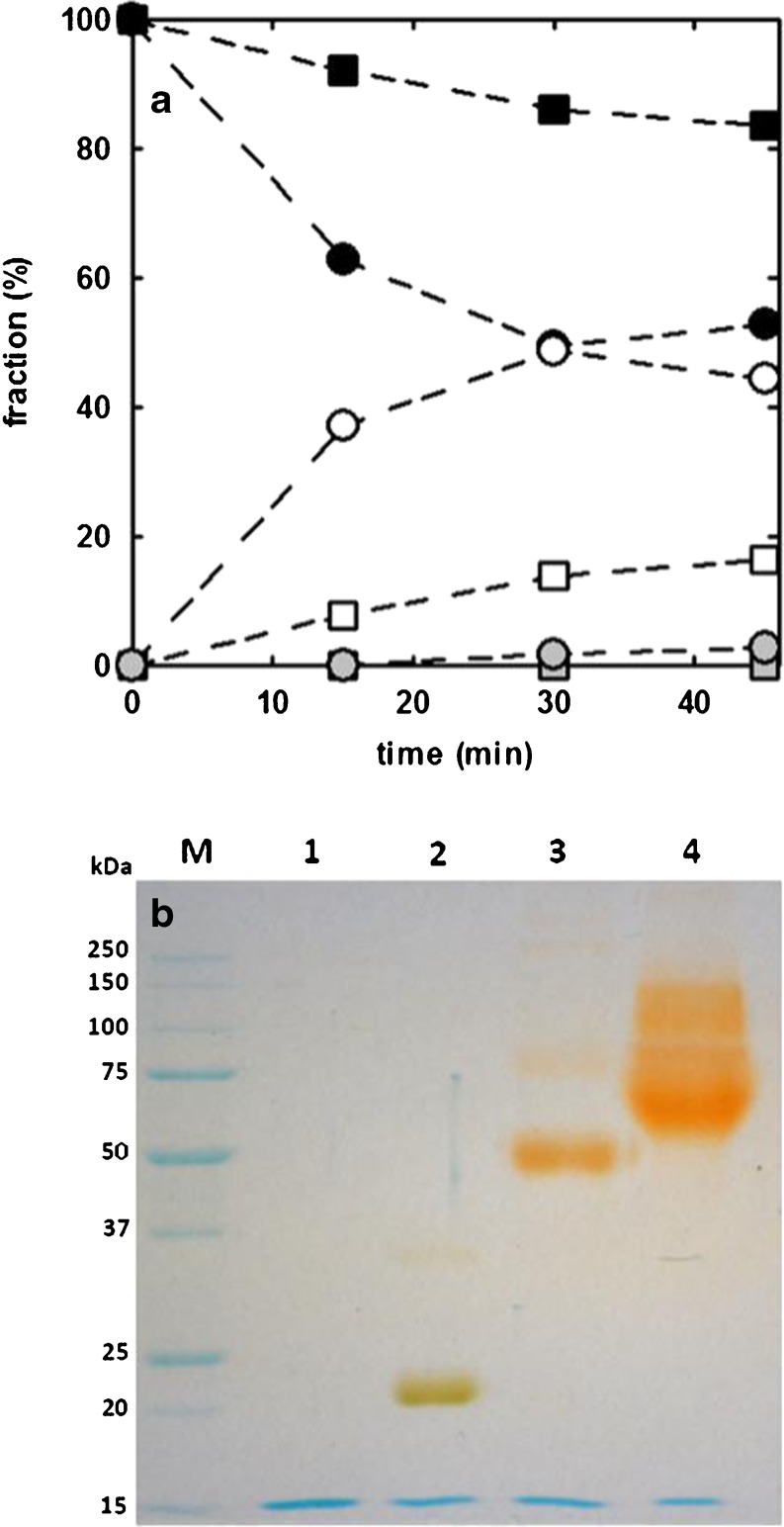
PEGylation of *Dr*Urah. Panel (**a**) PEGylation kinetics of *Dr*Urah at different PEG/cysteine molar ratios. The conjugation reaction was carried out at 20 °C, at a MAL-PEG/reactive cysteines molar ratio of 7.5 (*circles*) and 1.25 (*squares*). *Grey symbols* refer to the fraction of di-PEGylated monomers, open symbols refer to the fraction of mono-PEGylated monomers, and closed symbols to the fraction of unmodified monomers. *Dashed lines* through data points are just drawn for eye guidance. Panel (**b**) SDS-PAGE gel of unmodified and PEGylated *Dr*Urah (PEG/cysteine molar ratio of 7.5). *Lane M,* MW standards, *Lane 1,* unmodified *Dr*Urah, *Lane 2, Dr*Urah + 5 kDa PEG, *Lane 3, Dr*Urah + 20 KDa PEG, *Lane 4, Dr*Urah + 40 kDa PEG. The gel underwent a double staining procedure, first with Coomassie blue (*blue bands*), and then with the PEG-specific dye barium iodide (*yellow-brown bands*).

Zebrafish 5-hydroxyisourate hydrolase (*Dr*Urah) (16) is a tetrameric protein possessing 2 cysteines per monomer (8 per tetramer) not engaged in disulfide bridges, and hence available for electrophilic attack on thiol group. Similarly to *Dr*Uox, we first tried to define the optimal PEGylation parameters for *Dr*Urah by measuring, as a function of time, the degree of PEGylation upon reaction with 5, 20 or 40 kDa MAL-PEG, at a 1.25 or 7.5 PEG/free cysteines molar ratio. Figure 3a reports the PEGylation yield determined by densitometric analysis of the SDS-PAGE gel bands (Fig. S3).

As already observed with *Dr*Uox, also for *Dr*Urah a PEG/free cysteines molar ratio of 7.5 resulted in a degree of derivatization significantly higher than with a 1.25 ratio, and the reaction apparently reached completion within 30 min. Hence, in all following experiments *Dr*Urah was PEGylated at 20 °C at a PEG/cysteine ratio of 7.5. The reaction with 5, 20 or 40 kDa MAL-PEG always yielded a good degree of derivatization, showing the presence of mono- and di-PEGylated hydrolase monomers as shown by the SDS-PAGE (Fig. 3b). Following the same procedure reported above for *Dr*Uox, gel bands underwent densitometric analysis to evaluate the relative amount of unmodified, mono- and di-PEGylated monomers, as well as the expected average number of PEG chains conjugated to *Dr*Urah tetramers and the expected fraction of unmodified tetramers (Table II).

**Table II:** PEGylation Yield and Enzymatic Activity of *Dr*Urah after Reaction with MAL-PEG (PEG/Cysteine Molar Ratio of 7.5)

	% non-PEGylated monomers ^a^	% mono-PEGylated monomers ^a^	% di-PEGylated monomers ^a^	Average no. of PEG bound (per tetramer) ^b^	% unmodified tetramers ^b^	Specific enzymatic activity ^c^
No PEG	100	0	0	0	100	483.1 ± 113.6
5 kDa PEG	26	71	3	3.1	1	240.1 ± 20.1
20 kDa PEG	26	61	13	3.5	1	795.1 ± 134.5
40 kDa PEG	42	15	43	4.0	3	722.3 ± 102.0

^a^determined from densitometric analysis of SDS PAGE

^b^ calculated from combinatorial analysis

^c^ specific enzymatic activity is expressed as μmol min^−1^ mg^−1^ (mean ± standard error). The specific enzymatic activity of freshly prepared enzyme was 433.8 ± 48.6

Independent of the chain length, the average number of conjugated PEG molecules per tetramer resulted to be higher than in the case of *Dr*Uox, and the fraction of non-conjugated tetramers appeared to be almost negligible. Enzymatic activity was fully preserved or even increased. Increased activity of PEGylated enzymes, though apparently counterintuitive, can be rationalized, e.g., by taking into account quaternary stabilization of oligomeric proteins (43) or selective stabilization of active conformations.

#### PEGylation of DrUrad

**Fig. 4:**
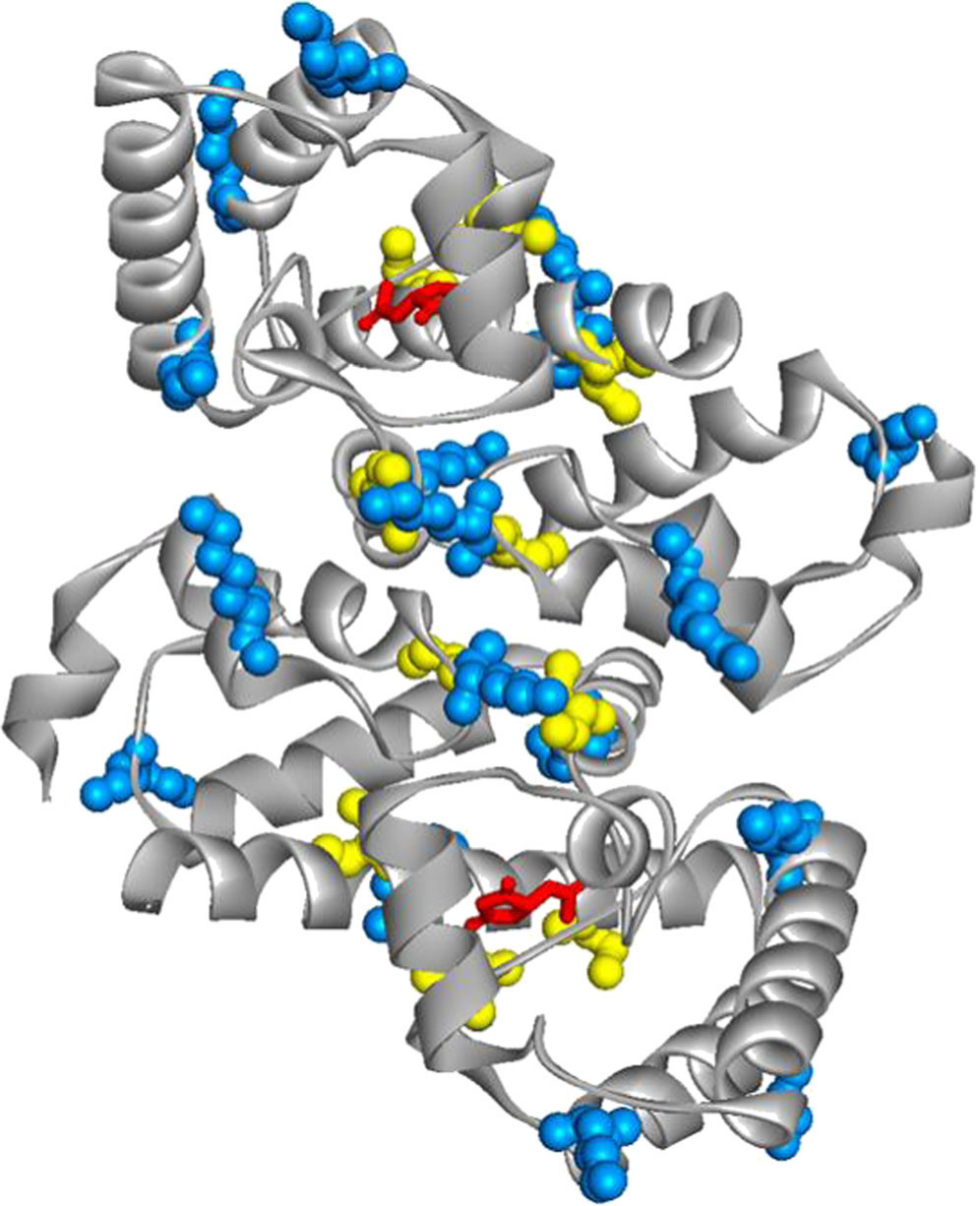
3D structure of dimeric *Dr*Urad (PDB ID: 2O73). Cysteine (*yellow*) and lysine (*light blue*) residues are shown in sphere representation. The (*S*)-allantoin molecule (*red*) bound to the active site is shown in ball and stick representation.

2-oxo-4-hydroxy-4-carboxy-5-ureidoimidazoline decarboxylase from zebrafish (*Dr*Urad) (15) is a homodimer with a large dimerization interface (2300 Å^2^/monomer) participating in both polar and apolar interactions (15). PEGylation with 5, 20 and 40 kDa MAL-PEG (7.5 PEG/free cysteines molar ratio, 30 min reaction time) occurred with good yields (Fig. S4), with an average of 1.8, 1.6 and 1.4 PEG chains bound per dimer, respectively. However, different from *Dr*Uox and *Dr*Urah, PEGylated *Dr*Urad was almost totally inactive, independent of the molecular weight of the PEG chain. Inspection of *Dr*Urad structure (15) indicates that of the five cysteine residues present in each monomer, two are at the dimer interface (Cys 25, and Cys 66) and one is close to the active site (Cys 122), although not directly involved in catalysis (Fig. 4).

Therefore, at least in principle, chemical derivatization of cysteine residues could interfere with *Dr*Urad function either by destabilizing the dimer interface, or by perturbing the accessibility of the active site. To discriminate between these alternatives, we first tried to protect cysteine residues from PEG conjugation by carrying out the PEGylation reaction in the presence of allantoin, the final product of *Dr*Urad catalysis, which binds to the active site as an inhibitor. Unfortunately, the binding of (*S*)-allantoin to the active site did not preserve enzymatic activity, resulting in a completely inactive PEGylated enzyme after removal of the inhibitor (data not shown).

We further investigated the role of Cys 25 and Cys 66 by exploiting their proximity, likely to favor the formation of a disulphide bridge under non-reducing conditions, as an alternative way to protect them from PEGylation. Incubation of *Dr*Urad at 20 °C for 5 h, corresponding to the time required for conjugation and dialysis of the PEGylated enzyme, in the absence of reducing agents results in an almost complete loss of enzymatic activity (Fig. S5). Such loss, that is not observed after incubation in ice for an equivalent time, is reverted after the addition of the reducing agents glutathione or tris(2-carboxyethyl)phosphine (TCEP) (Fig. S6). The reversible inactivation of *Dr*Urad was attributed to oxidation of cysteines and formation of disulphide bridges. Control SDS PAGE experiments in non reducing conditions upon protein alkylation are consistent with the formation of intra-subunit, but not inter-subunit bridges (data not shown). PEGylation trials carried out on inactive (oxidized) *Dr*Urad yielded an inactive enzyme even after the addition of reducing agents (data not shown). The fact that the reduction of oxidized cysteines is not sufficient for the recovery of the enzymatic activity indicates that chemical conjugation of residues involved in inter-subunit contacts is an important determinant of function loss upon PEGylation, likely because of dimer destabilization.

**Fig. 5:**
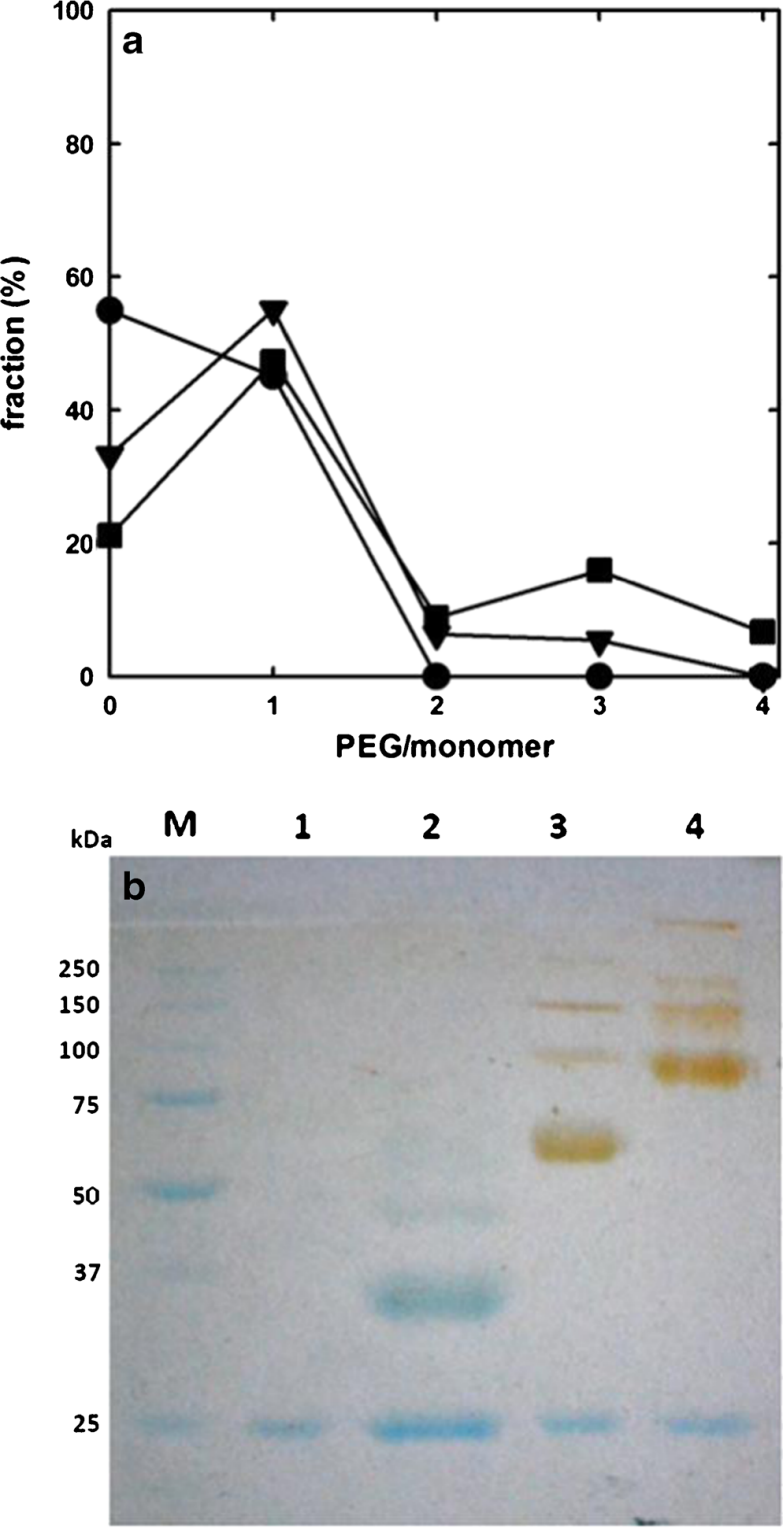
PEGylation of *Dr*Urad. Panel (**a**) Effect of MAL-PEG/*Dr*Urad ratio on the degree of PEGylation. Circles, triangles and squares refer to a MAL-PEG/*Dr*Urad ratio of 2.5, 5 and 10, respectively. Panel (**b**) SDS-PAGE gel of unmodified and PEGylated *Dr*Urad (MAL-PEG/*Dr*Urad molar ratio of 5). *Lane M,* MW standards, *Lane 1,* unmodified *Dr*Urad, *Lane 2, Dr*Urad + 5 kDa PEG, *Lane 3, Dr*Urad + 20 kDa PEG, *Lane 4, Dr*Urad + 40 kDa PEG. The gel underwent a double staining procedure, first with Coomassie blue (*blue bands*), and then with the PEG-specific dye barium iodide (*yellow-brown bands*).

Overall, our results led to dump maleimido-chemistry targeting cysteine side chains as a strategy for the obtainment of an active, PEGylated *Dr*Urad. In order to change the target aminoacid for the PEGylation reaction, while using the same PEG derivative (MAL-PEG), we took advantage of the extension arm-facilitated PEGylation, a two-step procedure which has already been used in PEGylation of human hemoglobin to increase the number of reaction sites (33,44) while avoiding the derivatization of cysteines with crucial role in protein activity (42,45). *Dr*Urad was pretreated with 2-iminothiolane (IMT), a cyclic molecule that reacts with the primary amino groups of lysine and N-terminal residues to form sulfhydryl groups. The opened IMT rings (extension arm) bearing a sulfhydryl group add additional, external reactive sites for the reaction with MAL-PEG, limiting the probability of reaction with cysteines. To further reduce the probability of possible reaction between cysteines and PEG, *Dr*Urad was incubated O/N at 15 °C to increase the thiol groups oxidation before PEGylation. Different experimental conditions were investigated, performing the reactions at 10, 5 or 2.5 MAL-PEG/*Dr*Urad molar ratio (Fig. S7), in the presence of a 20x or 40x IMT molar excess with respect to protein dimer concentration. The degree of PEGylation was evaluated by densitometric analysis of SDS PAGE bands. The molar excess of IMT had a scarce effect on the PEGylation yield (data not shown), since IMT is highly reactive and a 10x molar excess is enough to assure that most reactive amino groups are functionalized. Significant differences were observed between a MAL-PEG/*Dr*Urad ratio of 2.5 or 5, and much less between 5 and 10 (Fig. 5a).

Based on this result, and keeping in mind that an excessive PEGylation rate might involve cysteine side chains and prove detrimental for the maintenance of enzyme activity, *Dr*Urad was reacted with MAL-PEG of 5, 20 and 40 kDa molecular weight, keeping constant the 20x molar excess of IMT with respect to protein concentration and a MAL-PEG/*Dr*Urad molar ratio of 5. In all cases, the conjugation reaction resulted in a distribution mainly of unmodified and mono-PEGylated monomers, with smaller fractions of di-, tri- and tetra-PEGylated monomers (Fig. 5b). As previously reported for *Dr*Uox and *Dr*Urah, densitometric analysis of the SDS-PAGE bands (yielding the percentage of unmodified, mono-, di-, tri- and tetra-PEGylated monomers), and the subsequent combinatorial calculation (42), provided the expected average number of PEG chains conjugated to *Dr*Urad dimers and the expected fraction of unmodified dimers (Table III).

**Table III:** PEGylation Yield and Enzymatic Activity of *Dr*Urad after Reaction with MAL-PEG (MAL-PEG/*Dr*Urad Molar Ratio of 5)

	% non PEGylated monomers ^a^	% mono-PEGylated monomers ^a^	% di-PEGylated monomers ^a^	% tri-PEGylated monomers ^a^	% tetra-PEGylated monomers ^a^	Average no. of PEG bound (per dimer) ^b^	% Unmodified dimers ^b^	Specific enzymatic activity ^c^
No PEG	100	0	0	0	0	0	100	8.7 ± 0.3
5 kDa PEG	29	42	10	19	0	2.4	8	11.9 ± 0.4
20 kDa PEG	27	52	9	8	4	2.3	7	6.8 ± 0.4
40 kDa PEG	48	35	9	4	4	1.7	23	8.6 ± 0.3

^a^determined from densitometric analysis of SDS PAGE

^b^calculated rom combinatorial analysis

^c^ specific enzymatic activity is expressed as μmol min^−1^ mg^−1^ (mean ± standard error). The specific enzymatic activity of freshly prepared enzyme was 27 ± 0.7

In all cases, and at variance with respect to *Dr*Uox and *Dr*Urah, the activity of the PEGylated enzyme was significantly lower than the freshly prepared protein, though comparable to that of samples that underwent the same treatment, with the exclusion of MAL-PEG from the reaction solution. Thus, poor retention of catalytic activity appears to be related to protein processing rather than chemical conjugation with PEG. It has to be noted that data reported in Table III were originated by experiments carried out in the presence of reducing agents. The only partial retention of enzymatic activity of the unPEGylated protein does not contradict the full functional recovery induced by TCEP (Fig. S6), because the activities reported in Table III are from samples that underwent O/N dialysis, a procedure that is necessary to eliminate excess reagents, but is badly tolerated by *Dr*Urad (see also Materials and Methods). Indeed, when the dialysis procedure was replaced by a size-exclusion chromatography purification step, the activity of PEGylated *Dr*Urad was very close to that of the freshly prepared enzyme (Fig. S8). This suggest that the specific activity of a formulation containing PEGylated Uox, Urah and Urad might benefit of a SEC purification step of the enzymatic triad, or at least of the latter component. In the following, we preferred to apply the same protocol to the three enzymes, for the sake of simplicity and because the good catalytic efficiency of Urad might cope with partial loss of activity.

### Lyophilization of *Dr*Uox, *Dr*Urah and *Dr*Urad: Effect of PEG on Enzyme Activity and Stability

In view of defining a formulation for the three PEGylated enzymes for perspective use in the treatment of HPRT deficiency and other hyperuricemic conditions, lyophilization is the preferred strategy for storage, since it allows to produce a stable formulation, with long shelf life, in form of a powder to be resuspended at the moment of use. Therefore, for each enzyme, either unmodified or conjugated with 5, 20 or 40 kDa PEG, we evaluated the preservation of the enzymatic activity upon lyophilization and resuspension (after variable conservation times). Each sample was separately flash-frozen and lyophilized in the presence of 4% w/v mannitol and 1% w/v sucrose as lyoprotectants (35). After lyophilization, samples were stored at −20 °C. After resuspension in water for parenteral uses, enzymes samples were stored at 4 °C. The enzymatic activity of PEGylated *Dr*Uox, *Dr*Urah and *Dr*Urad was monitored for periods of up to several days and compared with that of the unmodified protein.

**Fig. 6:**
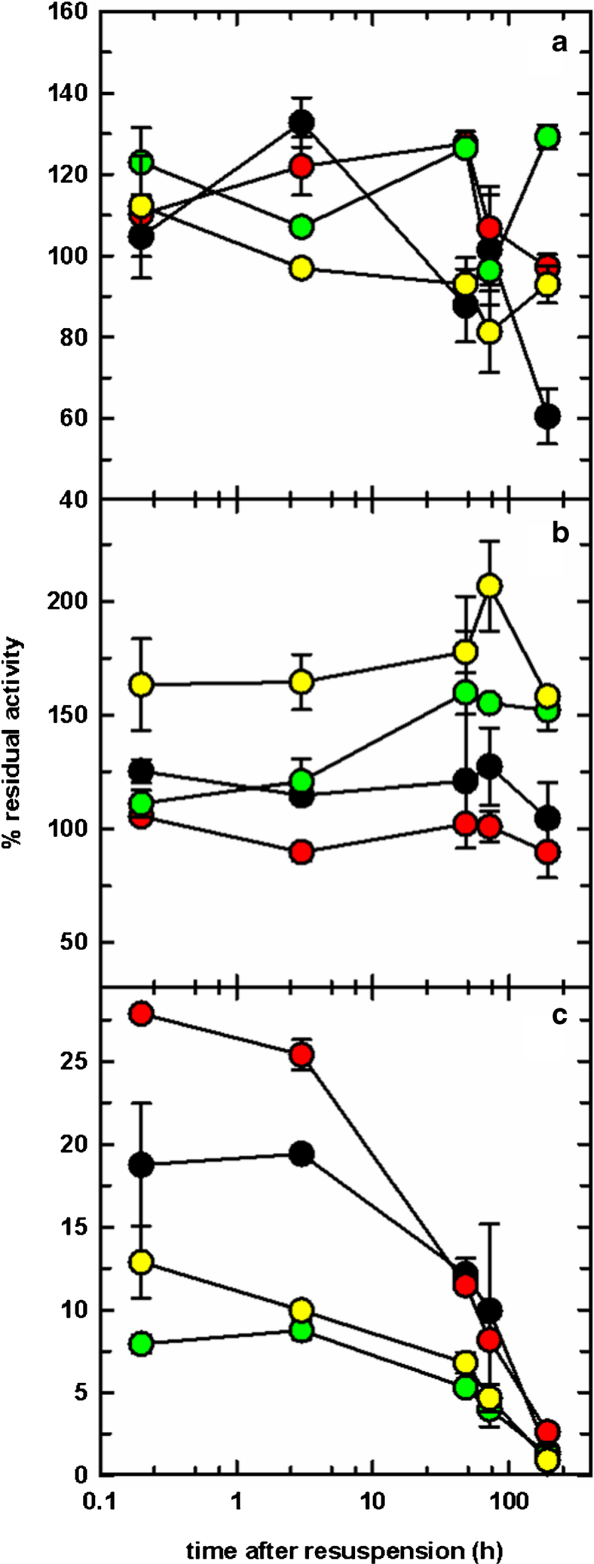
Activity of PEGylated enzymes after lyophilization and resuspension. Panel (**a**), *Dr*Uox; Panel (**b**), *Dr*Urah; Panel (**c**), *Dr*Urad. Different colors correspond to the different molecular weight of PEG conjugated to the enzymes: *black*, no PEG (control); *red*, 5 kDa PEG; green, 20 kDa PEG; yellow, 40 kDa PEG. Enzymatic activity is expressed as percentual mean ± standard error.

In the case of *Dr*Uox, PEGylation allows a higher retention of enzymatic activity, ranging between about 60–80% that of a freshly prepared, unmodified sample (Fig. 6a). Activity is virtually independent of the molecular weight of the conjugated chain, and is quantitatively maintained throughout a storage time after resuspension of 8 days at 4 °C.

An even better performance was shown by *Dr*Urah: all PEGylated forms exhibited a native-like enzymatic activity that was fully maintained for more than a week (Fig. 6b), with no significant differences related to PEG chain length.

In the case of *Dr*Urad, the initial enzymatic activity after lyophilization and resuspension was lower than that observed for the other two enzymes, and it gradually decayed to marginal values within 8 days storage at 4 °C (Fig. 6c). However, after 48 h still about half of the initial activity can be observed. PEG does not appear to confer increased stability upon long term storage.

### Catalytic Competence of a Combination of PEGylated *Dr*Uox, *Dr*Urah and *Dr*Urad and Comparison with DrUox Alone

The final goal of this work was to provide evidence that the three PEGylated enzymes *Dr*Uox, *Dr*Urah and *Dr*Urad conserve catalytic activity when co-present in the same reaction solution, so as to fully convert urate to (*S*)-allantoin with no accumulation of reaction intermediates.

It is hardly conceivable to set up a PEGylation protocol in which *Dr*Uox, *Dr*Urah and *Dr*Urad are mixed in appropriate molar ratios and PEGylated and lyophilized in a combined way, since each of the three enzymes has a different pool of reactive groups, each with specific reactivity. Therefore, in view of the preparation of a therapeutic formulation each enzyme will have to be processed separately, so that in principle PEG of different chain length can be used. However, since the results exposed in the previous sections indicate that in our experimental conditions the 20 kDa PEG is always a valuable solution (granting good retention of enzyme activity and low amounts of unreacted oligomers), we chose to test the *Dr*Uox-*Dr*Urah-*Dr*Urad combination using enzymes that had all been conjugated with 20 kDa PEG.

**Fig. 7:**
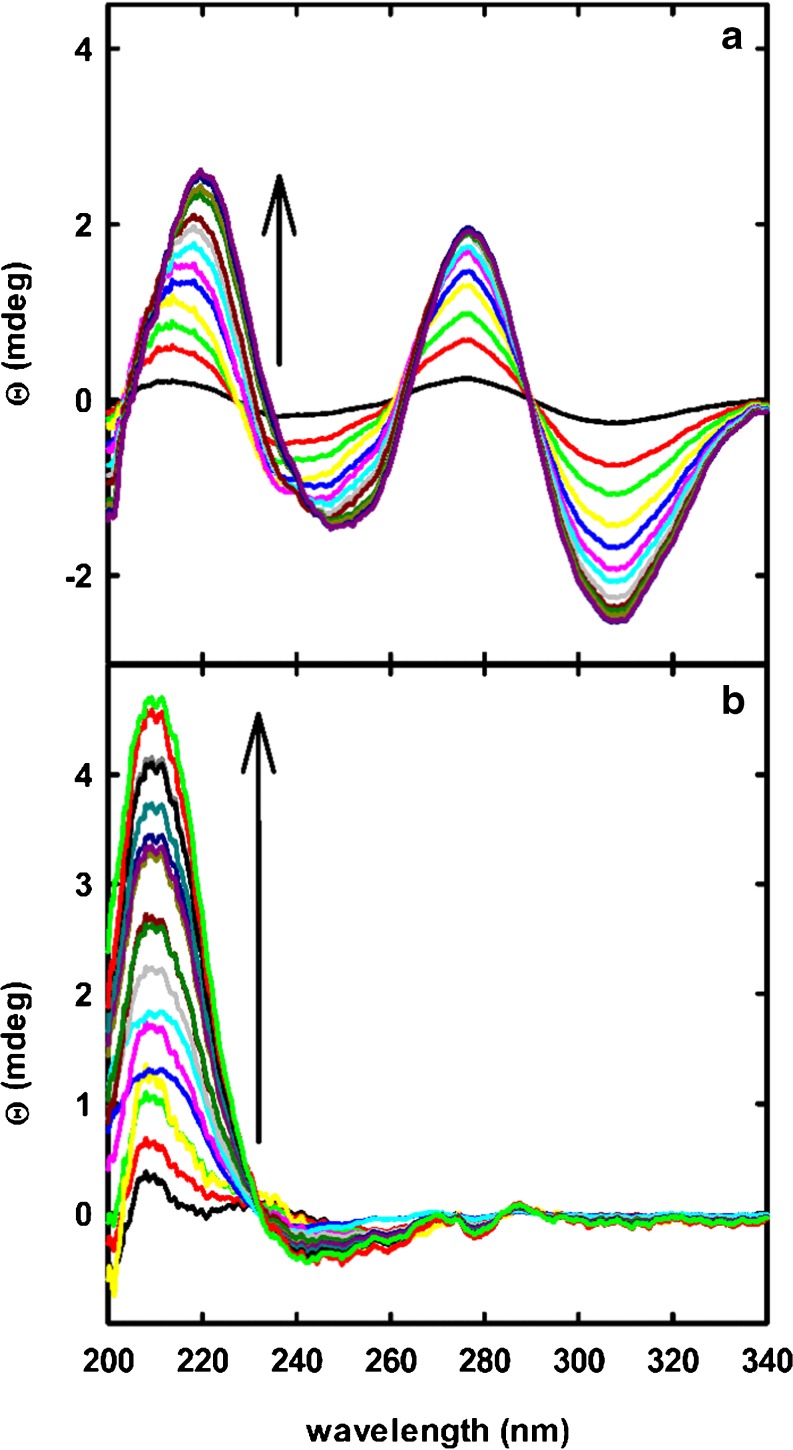
(**a**) Time-evolution of far-UV circular dichroism spectra of an urate solution in the presence of *Dr*Uox alone. Spectral time sequence (as indicated by *arrow*) of a solution containing 200 μM urate and 0.26 μM *Dr*Uox conjugated with 20 kDa PEG. Spectra were acquired every 5 min. (**b**) Time-evolution of far-UV circular dichroism spectra of an urate solution in the presence of *Dr*Uox, *Dr*Urah and *Dr*Urad. The solution contained 200 μM urate and 0.26 μM *Dr*Uox, 0.02 μM *Dr*Urah and 0.12 μM *Dr*Urad, all conjugated with 20 kDa PEG, in 20 mM potassium phosphate, pH 7.4. Spectra were acquired every 5 min. The time sequence of the spectra is indicated by the *arrow*.

To compare the efficacy of the enzyme triad or Uox alone in degrading uric acid *in vitro*, we exploited the different polarized light absorption properties of intermediate and final metabolites. Specifically, circular dichroism spectroscopy proved to be very sensitive to discriminate between HIU, OHCU and enantiomeric (*S*)-allantoin (3). Therefore, a solution containing 0.26 μM PEGylated *Dr*Uox was reacted with 200 μM urate -a concentration of the same order as the physiological range (46) -, and the reaction kinetic was followed by recording circular dichroism spectra as a function of time (Fig. 7).

The resulting spectra (Fig. 7a) showed the initial formation of positive peaks centered at 220 and 275 nm, and negative peaks at about 240 and 310 nm, which correspond to the spectrum of the (*S*)-HIU intermediate (3). At later times, a wavelength shift of the 220 and 240 peaks was observed. To identify the chemical species accumulating at longer times, a singular value decomposition (SVD) analysis of the kinetic series was carried out. This technique, besides wide application in noise reduction in spectroscopic data matrices (47), proved to be a powerful tool to extrapolate the spectra of intermediate species in kinetic series (48,49). The two principal spectral components identified by SVD analysis as accounting for the spectral changes observed in the kinetics are reported in Fig. S9. From comparison with spectra reported in literature (3), the two components can clearly be attributed to the HIU (black line) and OHCU (red line) intermediates. Therefore, at least under the reported experimental conditions the reaction of urate with the single enzyme *Dr*Uox brings to significant accumulation of HIU and OHCU, the former known to be a toxic metabolite in the mouse model (12).

On the contrary, when a solution containing PEGylated *Dr*Uox, *Dr*Urah and *Dr*Urad (0.26 μM, 0.02 μM, and 0.12 μM, respectively) was prepared, circular dichroism spectra after addition of urate to a 200 μM concentration clearly showed only the formation of a species with a positive peak at 208–210 nm and a less intense, broader negative band at around 240–250 nm (Fig. 7b).

These spectral features correspond to the *S* enantiomer of allantoin (3), demonstrating that in the presence of the three enzymes, urate is rapidly converted to the soluble metabolite (*S*)-allantoin with no accumulation of oxidized purines.

## Discussion

The goal of this work was to demonstrate that all three enzymes involved in the uricolytic degradation pathway of purine catabolism, whose evolutionary loss exposes humans to hyperuricemia-related diseases, can be obtained in a form suitable for therapy after recombinant expression and covalent conjugation with PEG.

Different strategies for protein PEGylation have been developed during the last decades, from the reaction of activated PEG (e.g. PEG aldehyde) with amino groups, such as the ε-amino group of lysines and N-terminal groups, to thiol derivatization of cysteines and enzymatic PEGylation using transglutaminase at glutamine or lysine residues (19–22,37). When PEGylation is applied for the development of therapeutics, purity and conjugation homogeneity are a major concern, consequently a chemistry that ensures site-selective conjugation and control over the degree of PEGylation is preferred because it favors activity retention and batch-to-batch reproducibility. Differently from the strategy applied with Pegloticase we did not pursue hyper-PEGylation. Although hyper-PEGylation could improve biocompatibility of non-human enzymes, it can also be expected that the higher is the degree of PEG derivatization, the higher is the heterogeneity of resulting chemical species, and the lower is enzyme activity. Considering also the existing claims that suggest the implication of the global size of the PEG conjugated complex (26) in determining unexpected fast clearance of conjugates, with highly PEGylated and large conjugates easily cleared, we decided to avoid hyper-PEGylation.

Based on the knowledge of the three-dimensional structure and catalysis of *Dr*Uox, *Dr*Urah, and *Dr*Urad, we decided to exploit reactive cysteine residues to obtain MAL-PEG derivatives; where cysteine derivatization caused a loss of activity (*Dr*Urad), an extension arm-facilitated PEGylation chemistry was used. The fraction of unPEGylated monomers observed for *Dr*Uox, *Dr*Urah, and *Dr*Urad under denaturing conditions (see Figs. 2, 3 and 5) is not of particular concern in view of the highly stable oligomeric organization exhibited by these proteins in solution. This ensures that in our PEGylation conditions only a minor fraction of proteins in their native assembly is present in an unmodified form. Among the different PEG lengths tested, the 20 kDa PEG appears to be a valuable solution for all three proteins, as it yielded a high fraction of conjugated oligomers (see Tables I, II, and III) and its linear size is suited for shielding the entire oligomer.

The main improvement of our enzymatic approach for management of uric acid levels, with respect to current standards, is in the lack of accumulation of intermediate urate oxidation products; this requires that urate oxidation is the rate limiting step of the overall reaction, and that the three enzymes involved in the pathway have stable and reproducible specific activity. In the case of *Dr*Uox and *Dr*Urah we observed excellent retention of the enzymatic activity of the PEGylated proteins, while *Dr*Urad was found to be the more labile component of the enzymatic triad. The propensity of *Dr*Urad to be inactivated by spontaneous oxidation might represent a serious issue in view of its administration as a protein therapeutic; however, this aspect will need to be evaluated by specific in vivo experiments, since plasma concentrations of reducing agents such as glutathione might be sufficient to maintain the protein in the fully active form. However, three critical points need be highlighted:

1. we defined PEGylation conditions compatible with the preservation of catalytic activity of each of the three enzymes involved in urate degradation pathway. Structural integrity of the proteins was checked by comparing secondary structure with that of their unPEGylated counterparts through far-UV circular dichroism spectra (Fig. S10);
2. lyophilization and resuspension of PEGylated proteins after storage at −20 °C do not significantly affect enzymatic activity. Even in the case of the less stable *Dr*Urad, the activity just after PEGylation (Table III) is comparable to that initially retrieved after resuspension of lyophilized samples (Fig. 6c);
3. despite the catalytic activity of *Dr*Urad appears to be less stable than *Dr*Uox and *Dr*Urah as a function of storage time, the lyophilized enzyme retains a measurable activity for at least a few days upon resuspension, and upon appropriate dosage can effectively complete the set of PEGylated proteins required for full enzymatic conversion of urate to soluble (*S*)-allantoin (Fig. 7b), in a formulation suitable for long storage and intravenous administration.

## 4. Conclusions

The current standard for therapy of hyperuricemia-related diseases considers either an uricostatic strategy, i.e. inhibition of xanthine oxidase to reduce uric acid production, or an uricolytic approach, exploiting intravenous administration of PEGylated Uox to favor the conversion of insoluble urate to HIU. The latter slowly converts to OHCU and racemic allantoin through spontaneous reactions. While uricostatic therapy causes the accumulation of upstream metabolites (allopurinol blocks the conversion of hypoxanthine into xanthine and of xanthine into uric acid), particularly when the HPRT activity is impaired, the main flaw of the uricolytic approach consists in the accumulation of the HIU intermediate. We suggest that the current therapeutic standard could be greatly improved if Urah and Urad, the two enzymes that complete the purine degradation pathway, could be administered in combination with Uox in a biocompatible and bioavailable form.

The present work shows that all the three enzymes that constitute the urate degradation pathway in most animals, and whose function is lost in hominoids, can be obtained in recombinant form and maintain an acceptable degree of activity after PEGylation, necessary to reduce immunogenicity and to increase circulation lifetimes, and lyophilization, granting long term storage of the formulation. A combination of the three enzymes, in an appropriate ratio, demonstrated to effectively convert uric acid to (*S*)-allantoin, with no accumulation of intermediate metabolites. These encouraging results pose the basis for future tests in a mouse model of disease that we are currently pursuing.

## Electronic supplementary material

DrUox, DrUrah and DrUrad structures and accessible surface area; SDS-PAGE of DrUox, DrUrah and DrUrad upon PEGylation, as a function of time and at different PEG/cys ratios; representative enzyme kinetics of DrUrad in the presence and in the absence of TCEP; relative enzymatic activity of DrUrad under oxidizing and reducing conditions; SDS-PAGE of DrUrad upon PEGylation with IMT pretreatment, at different MAL-PEG/protein ratios; relative enzyme activity after IMT reaction (DrUrad) and after purification by SEC of PEGyated samples (DrUox, DrUrah, DrUrad); principal components retrieved by SVD analysis of far-UV circular dichroism spectral changes of an urate solution in the presence of DrUox; far-uv circular dichroism spectra of PEGylated and unPEGylated DrUox, DrUrah and DrUrad.

ESM 1 (PDF 1.49 mb)

## Abbreviations

ASA: Accessible surface area
GMP: Guanosine monophosphate
HIU: 5-hydroxyisourate
HPRT: Hypoxanthine-guanine phosphoribosyltransferase
IMP: Inosine monophosphate
IMT: 2-iminothiolane
LND: Lesch-Nyhan disease
MAL: Maleimide
OHCU: 2-oxo-4-hydroxy-4-carboxy-5-ureidoimidazoline
PEG: Polyethylene glycol
PRPP: Phosphoribosyl pyrophosphate
TCEP: Tris(2-carboxyethyl)phosphine
Uox: Urate oxidase
Urad: OHCU decarboxylase
Urah: HIU hydrolase
