# Supplementary material for: A Trivalent Enzymatic System for Uricolytic Therapy of HPRT Deficiency and Lesch-Nyhan Disease
Source: Pharm Res. 2017 May 15;34(7):1477–90. doi: 10.1007/s11095-017-2167-6 (PMC5445154; doi:10.1007/s11095-017-2167-6)
Supplement: Supplementary file 1 — (PDF 1.49 mb) [file 11095_2017_2167_MOESM1_ESM.pdf]

SUPPLEMENTARY INFORMATION TO

A trivalent enzymatic system for the uricolytic therapy of HPRT deficiency and Lesch-Nyhan disease

Luca Ronda,[a] *Marialaura Marchetti,*[b] *Riccardo Piano,*[a] *Anastasia Liuzzi,*[b] *Romina Corsini,*[b] *Riccardo Percudani,**[b] *and Stefano Bettati**[a,c]

[a]Department of Medicine and Surgery, University of Parma, Parco Area delle Scienze 23/A, 43124 Parma, Italy

[b]Department of Chemistry, Life Sciences, and Environmental Sustainability, University of Parma, Parco Area delle Scienze 23/A, 43124 Parma, Italy

[c]National Institute of Biostructures and Biosystems, Viale Medaglie d'Oro 305, 00136, Rome, Italy

Running head: A triad of PEGylated enzymes for uricolysis

***Corresponding Authors**

Stefano Bettati
Department of Medicine and Surgery, University of Parma,
Parco Area delle Scienze 23/A, 43124, Parma Italy
E-mail: stefano.bettati@unipr.it;
Phone: +39 0521 905502; Fax: +39 0521 905151

Riccardo Percudani,
Department of Chemistry, Life Sciences,
and Environmental Sustainability, University of Parma,
Parco Area delle Scienze 23/A, 43124, Parma, Italy
E-mail: riccardo.percudani@unipr.it;

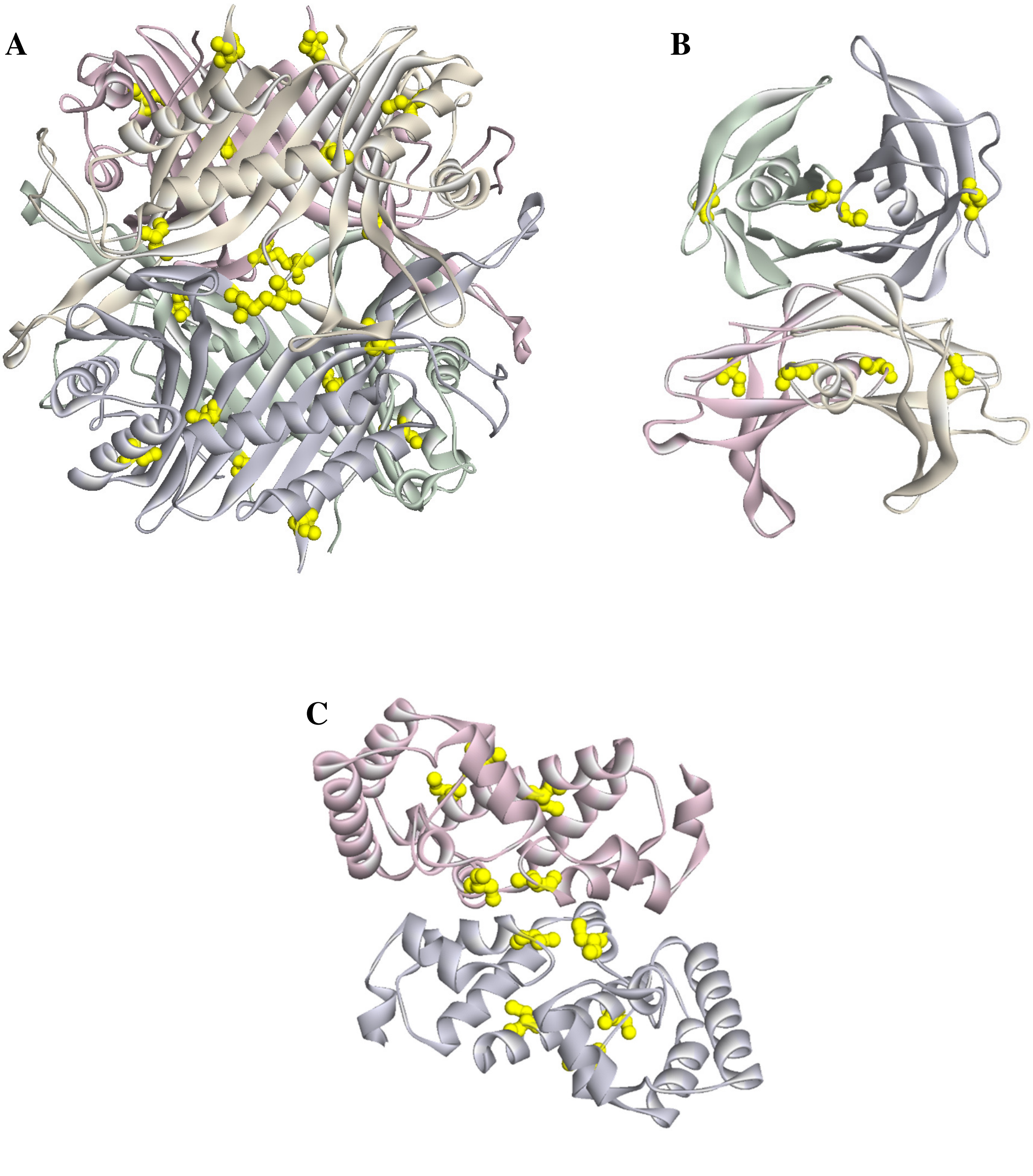

Figure S1. Quaternary organization and localization of cysteines in the structures of *Dr*Uox, *Dr*Urah and *Dr*Urad. Different monomers are rendered in different colors. Cysteines residues are shown in sphere representation and colored yellow. (A) *Dr*Uox (PDB ID: 5M98) (B) *Dr*Urah (PDB ID: 2H6U) (C) *Dr*Urad (PDB ID: 2O73).

Table S1. Accessible surface area (ASA) of lysine residues in *Dr*Uox, *Dr*Urah, and *Dr*Urad.

Enzyme	Chain	Residue	ASA	fASA
Uox (5M98)	A	18 LYS	3.7	0.02
Uox (5M98)	A	22 LYS	25.8	0.15
Uox (5M98)	A	47 LYS	131.9	0.75
Uox (5M98)	A	50 LYS	70	0.4
Uox (5M98)	A	67 LYS	41.8	0.24
Uox (5M98)	A	75 LYS	37	0.21
Uox (5M98)	A	77 LYS	94.8	0.54
Uox (5M98)	A	80 LYS	164.5	0.93
Uox (5M98)	A	104 LYS	55.7	0.32
Uox (5M98)	A	113 LYS	94	0.53
Uox (5M98)	A	117 LYS	79	0.45
Uox (5M98)	A	144 LYS	129	0.73
Uox (5M98)	A	153 LYS	58.3	0.33
Uox (5M98)	A	156 LYS	56.8	0.32
Uox (5M98)	A	159 LYS	1.9	0.01
Uox (5M98)	A	180 LYS	146.3	0.83
Uox (5M98)	A	206 LYS	130	0.74
Uox (5M98)	A	209 LYS	35.8	0.2
Uox (5M98)	A	215 LYS	35.8	0.2
Uox (5M98)	A	231 LYS	48.4	0.27
Uox (5M98)	A	266 LYS	161.2	0.91
Uox (5M98)	A	272 LYS	158	0.9
Uox (5M98)	A	293 LYS	99.3	0.56
Uox (5M98)	B	18 LYS	3.2	0.02
Uox (5M98)	B	22 LYS	32.1	0.18
Uox (5M98)	B	47 LYS	106	0.6
Uox (5M98)	B	50 LYS	70.8	0.4
Uox (5M98)	B	67 LYS	43.8	0.25
Uox (5M98)	B	75 LYS	39.7	0.22
Uox (5M98)	B	77 LYS	95.7	0.54
Uox (5M98)	B	80 LYS	162.9	0.92
Uox (5M98)	B	104 LYS	58.3	0.33
Uox (5M98)	B	113 LYS	120.1	0.68
Uox (5M98)	B	117 LYS	79.9	0.45
Uox (5M98)	B	144 LYS	134.5	0.76
Uox (5M98)	B	153 LYS	60.9	0.34
Uox (5M98)	B	156 LYS	56.9	0.32
Uox (5M98)	B	159 LYS	4.8	0.03
Uox (5M98)	B	180 LYS	132.9	0.75
Uox (5M98)	B	206 LYS	123.8	0.7
Uox (5M98)	B	209 LYS	24.7	0.14
Uox (5M98)	B	215 LYS	35.8	0.2
Uox (5M98)	B	231 LYS	57.2	0.32
Uox (5M98)	B	266 LYS	163.2	0.92
Uox (5M98)	B	272 LYS	149.5	0.85
Uox (5M98)	B	293 LYS	101.7	0.58
Uox (5M98)	C	18 LYS	3.8	0.02
Uox (5M98)	C	22 LYS	22.6	0.13
Uox (5M98)	C	47 LYS	127.5	0.72
Uox (5M98)	C	50 LYS	76	0.43
Uox (5M98)	C	67 LYS	43.9	0.25
Uox (5M98)	C	75 LYS	32.7	0.19
Uox (5M98)	C	77 LYS	92	0.52
Uox (5M98)	C	80 LYS	164.6	0.93
Uox (5M98)	C	104 LYS	56.3	0.32
Uox (5M98)	C	113 LYS	99.5	0.56
Uox (5M98)	C	117 LYS	87.4	0.5
Uox (5M98)	C	144 LYS	138.7	0.79
Uox (5M98)	C	153 LYS	57.8	0.33
Uox (5M98)	C	156 LYS	55.1	0.31
Uox (5M98)	C	159 LYS	2.8	0.02
Uox (5M98)	C	180 LYS	135.4	0.77
Uox (5M98)	C	206 LYS	124.9	0.71

Enzyme	Chain	Residue	ASA	fASA
Uox (5M98)	C	209 LYS	18.1	0.1
Uox (5M98)	C	215 LYS	33.7	0.19
Uox (5M98)	C	231 LYS	52.3	0.3
Uox (5M98)	C	266 LYS	156.6	0.89
Uox (5M98)	C	272 LYS	143	0.81
Uox (5M98)	C	293 LYS	106.3	0.6
Uox (5M98)	D	18 LYS	4.5	0.03
Uox (5M98)	D	22 LYS	27	0.15
Uox (5M98)	D	47 LYS	124.7	0.71
Uox (5M98)	D	50 LYS	63.7	0.36
Uox (5M98)	D	67 LYS	41.5	0.23
Uox (5M98)	D	75 LYS	38.2	0.22
Uox (5M98)	D	77 LYS	88.6	0.5
Uox (5M98)	D	80 LYS	142	0.8
Uox (5M98)	D	104 LYS	52.1	0.3
Uox (5M98)	D	113 LYS	92.3	0.52
Uox (5M98)	D	117 LYS	113.9	0.65
Uox (5M98)	D	144 LYS	125.1	0.71
Uox (5M98)	D	153 LYS	59.9	0.34
Uox (5M98)	D	156 LYS	54.7	0.31
Uox (5M98)	D	159 LYS	4	0.02
Uox (5M98)	D	180 LYS	152.5	0.86
Uox (5M98)	D	206 LYS	127.5	0.72
Uox (5M98)	D	209 LYS	30.5	0.17
Uox (5M98)	D	215 LYS	33.1	0.19
Uox (5M98)	D	231 LYS	46.5	0.26
Uox (5M98)	D	266 LYS	144.1	0.82
Uox (5M98)	D	272 LYS	168.5	0.95
Uox (5M98)	D	293 LYS	104.2	0.59
Urah (2H6U)	A	59 LYS	114.3	0.65
Urah (2H6U)	A	68 LYS	21.7	0.12
Urah (2H6U)	A	75 LYS	138.6	0.79
Urah (2H6U)	B	59 LYS	105.1	0.6
Urah (2H6U)	B	68 LYS	19.8	0.11
Urah (2H6U)	B	75 LYS	136.3	0.77
Urah (2H6U)	C	59 LYS	104.3	0.59
Urah (2H6U)	C	68 LYS	21.4	0.12
Urah (2H6U)	C	75 LYS	138.8	0.79
Urah (2H6U)	D	59 LYS	112.5	0.64
Urah (2H6U)	D	68 LYS	21.3	0.12
Urah (2H6U)	D	75 LYS	132.8	0.75
Urad (2O73)	A	16 LYS	142.6	0.81
Urad (2O73)	A	24 LYS	74.1	0.42
Urad (2O73)	A	39 LYS	157.2	0.89
Urad (2O73)	A	60 LYS	25.1	0.14
Urad (2O73)	A	112 LYS	35.5	0.2
Urad (2O73)	A	128 LYS	83	0.47
Urad (2O73)	A	140 LYS	124	0.7
Urad (2O73)	A	155 LYS	40.1	0.23
Urad (2O73)	A	156 LYS	92.1	0.52
Urad (2O73)	B	16 LYS	107.3	0.61
Urad (2O73)	B	24 LYS	84	0.48
Urad (2O73)	B	39 LYS	155.7	0.88
Urad (2O73)	B	60 LYS	20.3	0.11
Urad (2O73)	B	112 LYS	44.4	0.25
Urad (2O73)	B	128 LYS	73.9	0.42
Urad (2O73)	B	140 LYS	147.1	0.83
Urad (2O73)	B	155 LYS	36.1	0.2
Urad (2O73)	B	156 LYS	87.5	0.5

ASA analysis was performed with the Vadar web server (http://vadar.wishartlab.com/); buried residues (fASA<5%) are shaded in gray.

Table S2. Accessible surface area (ASA) of cysteine residues in *Dr*Uox, *Dr*Urah, and *Dr*Urad.

Enzyme	Chain	Residue	ASA	fASA
Uox (5M98)	A	90 CYS	0	0
Uox (5M98)	A	129 CYS	3.7	0.04
Uox (5M98)	A	136 CYS	0	0
Uox (5M98)	A	185 CYS	0	0
Uox (5M98)	A	291 CYS	15.2	0.14
Uox (5M98)	B	90 CYS	0	0
Uox (5M98)	B	129 CYS	1.4	0.01
Uox (5M98)	B	136 CYS	0.3	0
Uox (5M98)	B	185 CYS	0	0
Uox (5M98)	B	291 CYS	14.2	0.13
Uox (5M98)	C	90 CYS	0	0
Uox (5M98)	C	129 CYS	2.5	0.02
Uox (5M98)	C	136 CYS	0.2	0
Uox (5M98)	C	185 CYS	0.3	0
Uox (5M98)	C	291 CYS	16.3	0.15
Uox (5M98)	D	90 CYS	0	0
Uox (5M98)	D	129 CYS	0.6	0.01
Uox (5M98)	D	136 CYS	0.2	0
Uox (5M98)	D	185 CYS	0	0
Uox (5M98)	D	291 CYS	16.8	0.16

Enzyme	Chain	Residue	ASA	fASA
Urah (2H6U)	A	53 CYS	4.9	0.05
Urah (2H6U)	A	84 CYS	0.1	0
Urah (2H6U)	B	53 CYS	5.7	0.05
Urah (2H6U)	B	84 CYS	0.2	0
Urah (2H6U)	C	53 CYS	5	0.05
Urah (2H6U)	C	84 CYS	0.7	0.01
Urah (2H6U)	D	53 CYS	7.1	0.07
Urah (2H6U)	D	84 CYS	0.6	0.01
Urad (2O73)	A	25 CYS	0.5	0
Urad (2O73)	A	66 CYS	4	0.04
Urad (2O73)	A	122 CYS	2	0.02
Urad (2O73)	A	149 CYS	41.2	0.39
Urad (2O73)	A	158 CYS	2.8	0.03
Urad (2O73)	B	25 CYS	0.5	0
Urad (2O73)	B	66 CYS	4.8	0.04
Urad (2O73)	B	122 CYS	0.9	0.01
Urad (2O73)	B	149 CYS	41.3	0.39
Urad (2O73)	B	158 CYS	2.9	0.03

ASA analysis was performed with the Vadar web server (http://vadar.wishartlab.com/); buried residues (fASA<5%) are shaded in gray.

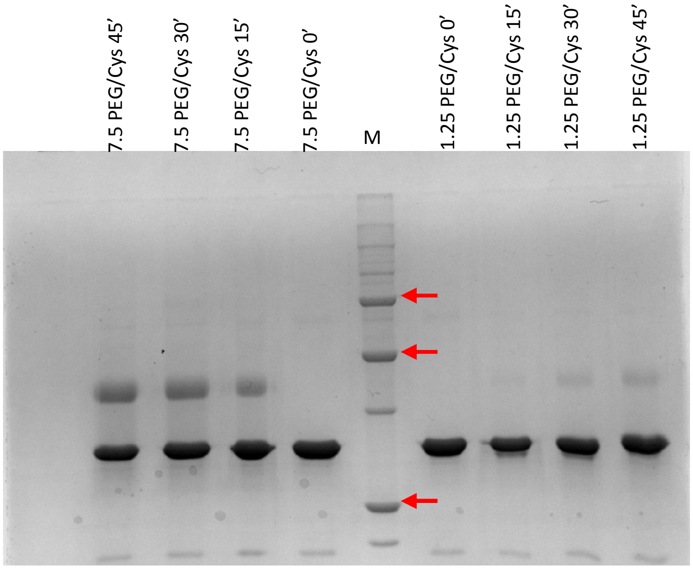

Figure S2. SDS PAGE of *Dr*Uox PEGylation as a function of time at two different MAL-PEG/free cysteine ratios. Unstained Precision Plus Protein(R) standards (Biorad) were used as MW markers (M); the bands corresponding to 25, 50 and 75 kDa are highlighted in the gel. In all other lanes, the most intense band at about 34 kDa corresponds to unPEGylated DrUox monomers.

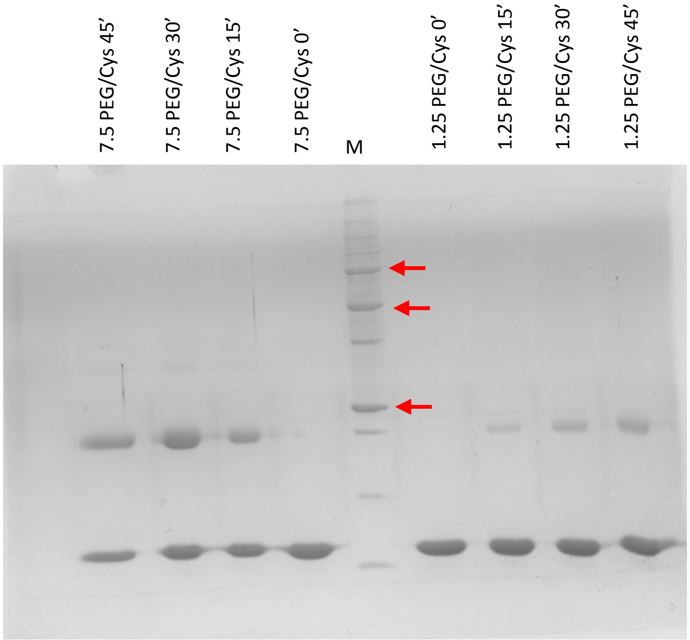

Figure S3. SDS PAGE of *Dr*Urah PEGylation as a function of time at two different MAL-PEG/free cysteine ratios. Unstained Precision Plus Protein® standards (Biorad) were used as MW markers (M) (25, 50 and 75 kDa bands are highlighted in the gel) and appear in lane 5. In all other lanes, the most intense band at about 13 kDa corresponds to unPEGylated *Dr*Urah monomers.

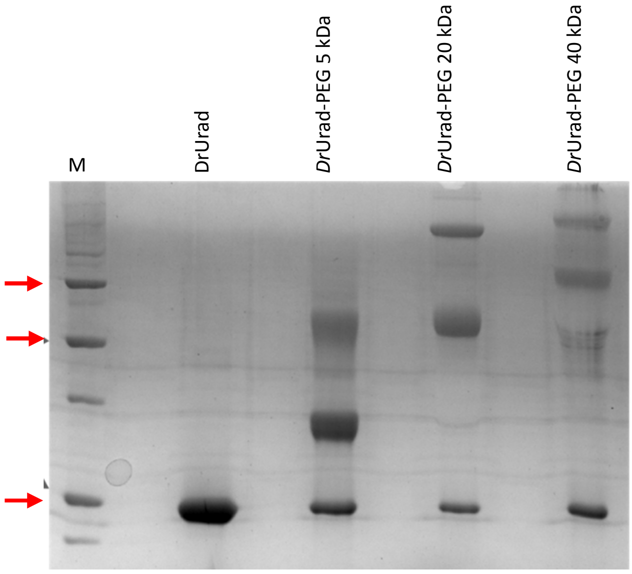

Figure S4. SDS-PAGE of *Dr*Urad before and after reaction with 5, 20 and 40 kDa MAL-PEG. The reaction was carried out at a PEG/cysteine ratio of 7.5, at 20 °C for 30 minutes. Unstained Precision Plus Protein(R) standards (Biorad) were used as MW markers (M) (25, 50 and 75 kDa bands are highlighted in the gel) and are shown in lane 1. In all other lanes, the band at about 22 kDa corresponds to unPEGylated *Dr*Urad monomers.

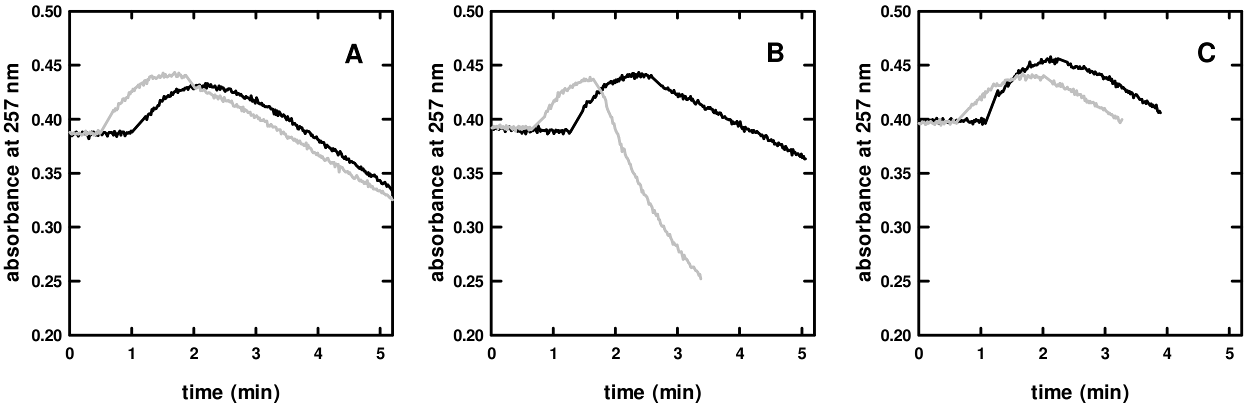

Figure S5. Example decay kinetics of 2-oxo-4-hydroxy-4-carboxy-5-ureidoimidazoline (OHCU) in the absence (black lines) and presence (grey lines) of tris(2-carboxyethyl)phosphine (TCEP). Decay kinetics are recorded by measuring the time evolution of light absorbance at 257 nm, the absorption peak of the Urad substrate OHCU. A: spontaneous decay; B: in the presence of unPEGylated *Dr*Urad; C: in the presence of PEGylated *Dr*Urad (5 kDa PEG, derivatization of Cys residues).

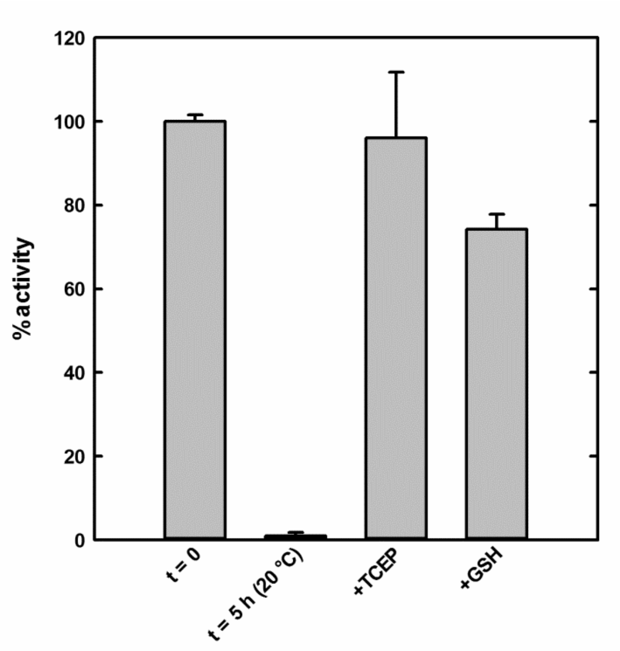

Figure S6. Relative enzymatic activity of *Dr*Urad under oxidizing and reducing conditions. From left to right, bars refer to freshly prepared *Dr*Urad (chosen as a reference), the enzyme spontaneously oxidized after an incubation of 5 hours at 20 °C, and the recovery of activity after treatment of the inactivated (oxidized) enzyme with 1 mM tris(2-carboxyethyl)phosphine (TCEP) or 1 mM glutathione (GSH).

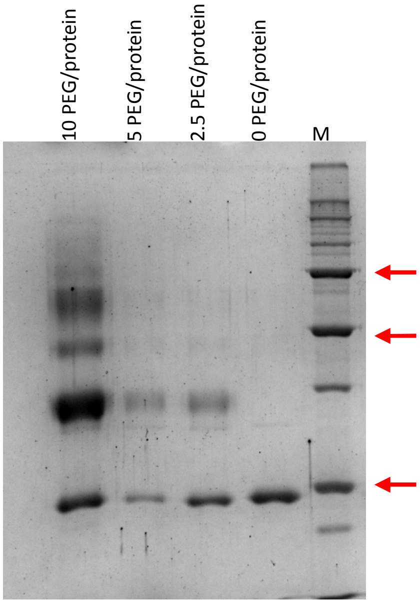

Figure S7. SDS-PAGE of *Dr*Urad PEGylation after reaction with iminothiolane. PEGylation reactions were carried out at an IMT/protein ratio of 20, at different MAL-PEG/protein ratios (2.5, 5 and 10). Unstained Precision Plus Protein(R) standards (Biorad) were used as MW markers (M) (25, 50 and 75 kDa bands are highlighted in the gel, lane 5).

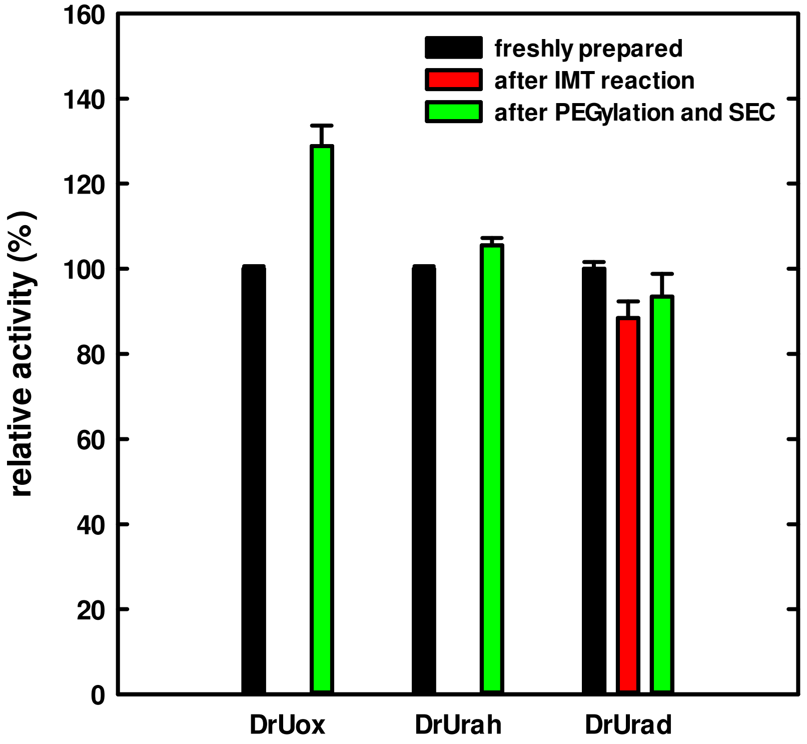

Figure S8. Relative enzyme activity after IMT reaction, or after PEGylation and SEC. Size-exclusion chromatography (SEC) was carried out in 100 mM potassium phosphate, 150 mM NaCl, pH 7.4, loading 10 µg of protein on a Superdex 200 Increase 3.2/300 (GE Healthcare), 0.1 mL/min. The enzymatic activity of freshly prepared *Dr*Uox, *Dr*Urah and *Dr*Urad was set as a reference (100%). The other columns show the relative activity of *Dr*Urad after reaction with IMT at an IMT/protein ratio of 20 (and before MAL-PEG reaction), and of all three enzymes after a size-exclusion chromatography purification step following conjugation with 20 kDa PEG, showing full retention of enzymatic activity upon PEGylation.

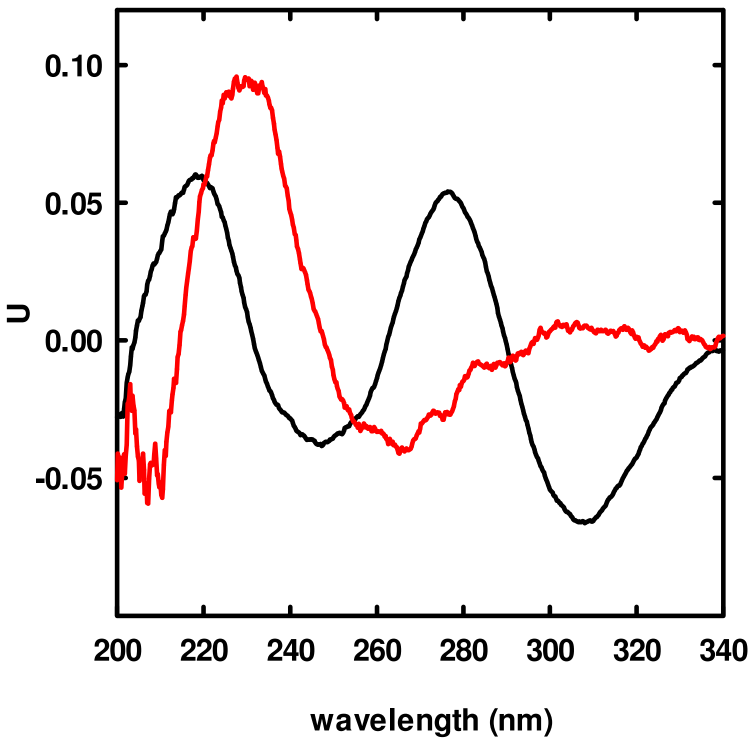

Figure S9. SVD Analysis. Principal components retrieved by SVD analysis of the observed far-UV circular dichroism spectral changes of a urate solution in the presence of *Dr*Uox alone (analysis from data in Figure 7A).

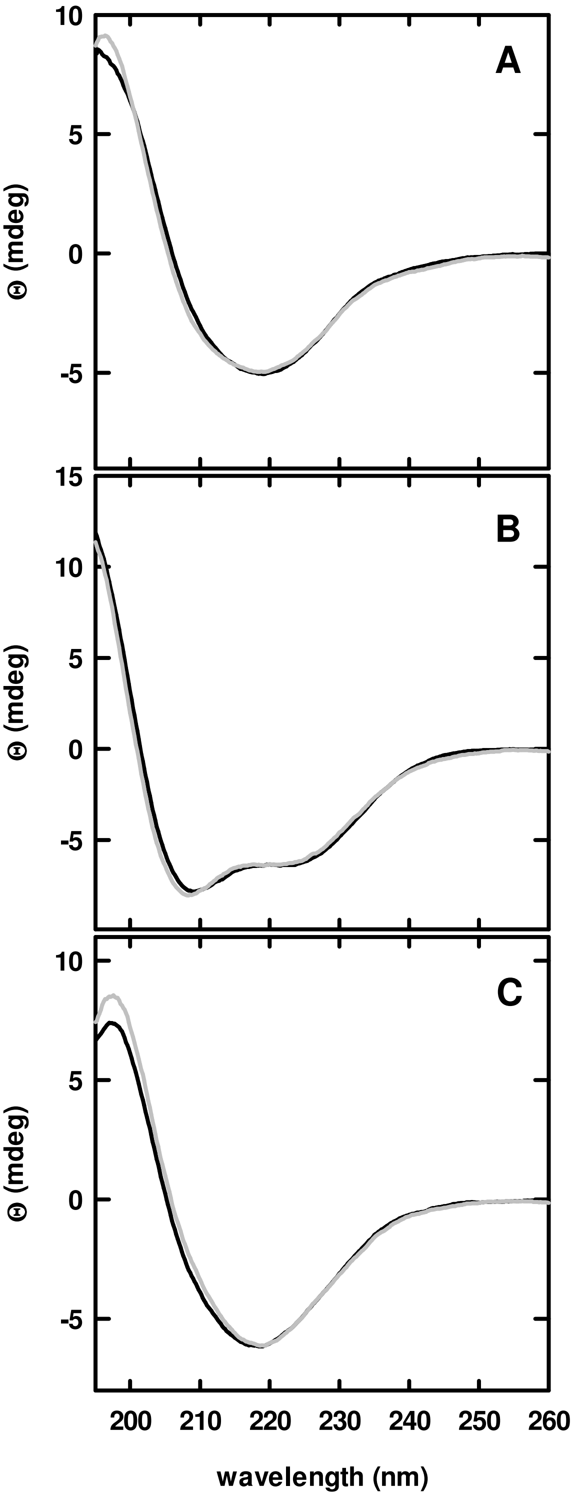

Figure S10. Circular dichroism spectra of un-PEGylated and PEGylated *Dr*Uox, *Dr*Urah and *Dr*Urad. Black lines: PEGylated proteins; grey lines: non-PEGylated proteins. Panel A: *Dr*Uox; Panel B: *Dr*Urah; Panel C: *Dr*Urad. Protein concentration was 2.2 µM, 5.1 µM and 3.0 µM, respectively, in 20 mM potassium phosphate, pH 7.4.
